# A Comprehensive Review of the Triangular Relationship among Diet–Gut Microbiota–Inflammation

**DOI:** 10.3390/ijms25179366

**Published:** 2024-08-29

**Authors:** Nidesha Randeni, Matteo Bordiga, Baojun Xu

**Affiliations:** 1 Food Science and Technology Program, Department of Life Sciences, BNU-HKBU United International College, Zhuhai 519087, China; nidesha.randeni96@gmail.com; 2 Department of Agricultural and Plantation Engineering, Faculty of Engineering Technology, The Open University of Sri Lanka, Nawala, Nugegoda 10250, Sri Lanka; 3 Department of Pharmaceutical Sciences, Università degli Studi del Piemonte Orientale “A. Avogadro”, Largo Donegani 2, 28100 Novara, Italy; matteo.bordiga@uniupo.it

**Keywords:** gut microbiota, inflammation, diet, dysbiosis, metabolites

## Abstract

The human gastrointestinal tract hosts a complex and dynamic community of microorganisms known as the gut microbiota, which play a pivotal role in numerous physiological processes, including digestion, metabolism, and immune function. Recent research has highlighted the significant impact of diet on the gut microbiota composition and functionality, and the consequential effects on host health. Concurrently, there is growing evidence linking the gut microbiota to inflammation, a key factor in many chronic diseases such as inflammatory bowel disease (IBD), obesity, diabetes, and cardiovascular diseases (CVDs). This review explores how dietary components influence the gut microbiota composition, how these microbial changes affect inflammatory pathways, and the therapeutic implications of modulating this axis for chronic inflammatory disease prevention and management. Beneficial dietary patterns, such as the Mediterranean diet (MD) and plant-based diets, promote a diverse and balanced gut microbiota composition, supporting anti-inflammatory pathways. Conversely, the Western diet (WD), high in saturated fats and refined sugars, is associated with dysbiosis and increased inflammation. With all the links between the three variables considered, this review attempts to offer a thorough examination of the triangle formed by inflammation, the gut microbiota, and food.

## 1. Introduction

The human gastrointestinal tract is home to a complex and dynamic community of microorganisms known as the gut microbiota. This intricate ecosystem plays a critical role in numerous physiological processes, including digestion, metabolism, immune function, and even behavior [1]. The composition and functionality of the gut microbiota are profoundly influenced by diet, with different dietary patterns inducing significant changes in microbial communities. For instance, diets rich in fiber, polyphenols, and healthy fats, such as the MD, promote the growth of beneficial bacteria and enhance microbial diversity. Conversely, the WD, characterized by a high intake of fats and refined sugars, is associated with decreased microbial diversity, an increase in pathogenic bacteria [2], and an increase in the number of mucin-degrading bacteria, which, in turn, can affect the intestinal mucus layer thickness [3].

In recent years, a growing body of evidence has emerged linking the gut microbiota to inflammation, which is a key factor in the pathogenesis of many chronic diseases, including IBD, obesity, diabetes, and CVDs [4]. The gut microbiota modulate the host’s immune system and inflammatory responses through various mechanisms. Beneficial bacteria help to maintain gut barrier integrity, preventing the translocation of harmful pathogens and toxins into the bloodstream [5]. They also produce metabolites, such as short-chain fatty acids (SCFAs), which have anti-inflammatory effects and support immune homeostasis [6]. However, an imbalance in the gut microbiota, known as dysbiosis, can disrupt these protective mechanisms, leading to increased gut permeability, systemic inflammation [7], and impaired intestinal barrier structure and function [3]. Understanding the triangular relationship among diet, the gut microbiota, and inflammation is crucial for developing effective strategies to improve health outcomes. The interplay between these elements is mediated through several mechanistic pathways. Dietary components directly influence the composition and activity of the gut microbiota [8], which, in turn, produce metabolites that can modulate immune responses and inflammation. For instance, SCFAs produced from the fermentation of dietary fiber enhance the gut barrier function and regulate inflammatory pathways, while diets high in saturated fats can promote the growth of pro-inflammatory bacteria and increase endotoxin levels, leading to systemic inflammation [9].

This review aims to synthesize the current knowledge on the diet–gut microbiota–inflammation axis, providing insights into how dietary interventions can modulate the gut microbiota composition and function, and, consequently, influence inflammatory processes. By exploring the mechanistic pathways and therapeutic potential of this interconnected triad, we hope to contribute to the development of effective strategies for the prevention and management of chronic inflammatory diseases.

## 2. Gut Microbiome: General Concepts of Diet and Gut Microbiota

The microbial community of the gut microbiome is extremely complicated. While there are diverse microbial communities in more proximal parts of the gastrointestinal tract, the colon has the highest biomass [10]. The human gut contains 10 times the number of microbial cells of the human body as a whole; these bacteria weigh about 2 kg, represent up to 5000 different species, and total about 100 trillion [11,12]. The composition of the gut microbiota comprises fungus, bacteria, viruses, and parasites [1,11]. Moreover, *Prevotella*, *Ruminococcus*, Bacteroidetes, and Firmicutes are among the most prominent kinds of bacteria [13]. Firmicutes are the most prevalent in the average adult, followed by Bacteroidetes and Actinobacteria [14]. *Eubacterium*, *Ruminococcus*, and *Clostridium* are the three subgroups of Firmicutes. It has been demonstrated that the proportion of the bacterial species Firmicutes to Bacteroidetes (F/B) affects both health and disease [15]. There is mounting evidence that suggests that nonbacterial components may also be involved in health and disease, despite the majority of studies being on bacteria [16]. The microbiota inoculate humans for the first time during birth. It has been demonstrated that a variety of factors, including the method of delivery (vaginal versus caesarian section), food (breast versus formula feeding), and use of antibiotics, affect the composition of an infant’s gut microbiota [17]. During the first few years of life, the microbiota quickly diversify [18]. They also play a significant role in the development of the immune system [19], providing defense against pathogenic microorganisms and bacterial overgrowth. In adults, the microbiota play a role in a number of processes, including bone density modulation [20], the modification and elimination of particular toxins and medications [21], and the optimization of intestinal barrier function [22]. The gut microbiome’s bacteria are involved in producing neurotransmitters like serotonin, vitamins, and enzymes, as well as obtaining energy from meals and maintaining a balance between an opportunistic and helpful bacterial makeup. For example, the bacterially generated vitamin K is involved in immunological and metabolic processes. Therefore, disease may arise from an imbalance in bacterial species [23]. Numerous factors, including the host genetics and physiology, environmental exposures, age, and food, influence the makeup and function of the microbiota [24]. A shift in diet composition can have an impact on this ratio, because nutrition has been shown to be one of the factors that can change the bacterial composition the most [11]. The microbiota respond quickly (within 24 h) to changes in diet in both people and mice [25].

## 3. Diet and Gut Microbiota

### 3.1. Subsection Role of Diet in Shaping Gut Microbiota

Diet refers to the sum of foods and beverages consumed by an individual or group, playing a crucial role in maintaining health and well-being. It provides the essential nutrients required for various bodily functions, including energy production, growth, repair, and the maintenance of physiological processes. The composition of one’s diet—encompassing macronutrients like carbohydrates, proteins, and fats, as well as micronutrients such as vitamins and minerals—significantly influences overall health outcomes [26].

Beyond providing essential nutrients, diet also has a profound impact on the gut microbiota, the diverse community of microorganisms living in the gastrointestinal tract. These microorganisms play key roles in digestion, immune function, and the production of essential metabolites. The interaction between diet and the gut microbiota is bidirectional: while diet shapes the composition and activity of the gut microbiota, the microbiota influence how nutrients and other dietary components are metabolized [27].

Different dietary patterns significantly influence health outcomes, particularly through their impact on the gut microbiota and inflammation [28]. The WD, characterized by high intakes of processed foods, red meats, sugars, and unhealthy fats, is often associated with adverse health effects, including a reduced microbial diversity and increased inflammation [29]. In contrast, the MD, rich in fruits, vegetables, whole grains, nuts, and olive oil, promotes a diverse and beneficial gut microbiota composition, contributing to anti-inflammatory effects and improved metabolic health [30]. Similarly, vegetarian and vegan diets, which emphasize plant-based foods, enhance gut health by increasing the abundance of fiber-fermenting bacteria and the production of anti-inflammatory metabolites [31]. Each of these dietary patterns offers unique insights into how dietary choices can shape the gut microbiota and influence inflammatory processes, thereby affecting overall health.

### 3.2. Effects of Dietary Patterns on Gut Microbiota

#### 3.2.1. Mediterranean Diet

**Figure 1:**
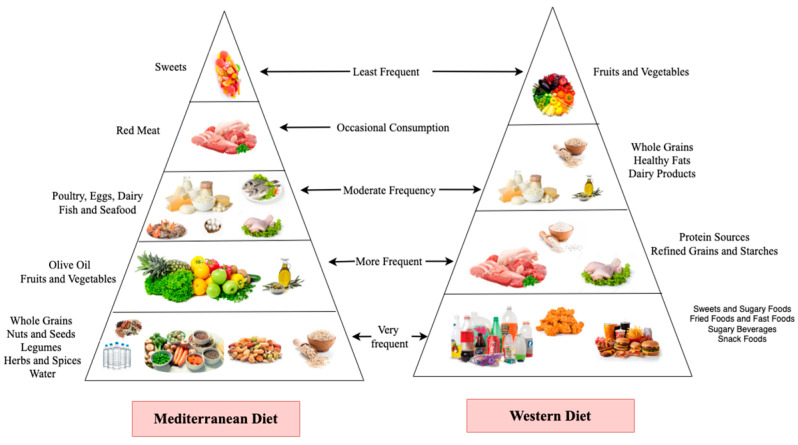
Mediterranean diet and Western diet.

A varied diet rich in nuts, fruits, vegetables, and olive oils, with moderate fish, poultry, and wine intake and a minimal intake of processed and red meats, is the hallmark of the MD [32] (Figure 1). Dietary fiber content is high in multiple components. Because of its numerous food groups, ease of accessibility, and health advantages, it is becoming more and more popular among medical and non-medical personnel. The MD is the most recommended plant-based dietary pattern, with an abundance of evidence to support it. High levels of MD adherence have also been positively linked to changes in the microbiota and their metabolites [33], pointing to a possible role for the gut microbiota in the advantageous impacts of this dietary strategy.

Diverse effects have been observed in human research models investigating the influence of the MD on the gut microbiome. Numerous studies have reported increased *Bifidobacterium* abundance. *Prevotella*, *Bacteroides*, and *Enterococcus* were also elevated in one of these trials [34]. *Faecalibacterium prausnitzii*, *Roseburia*, and *Lachnospiraceae* were found to be more abundant in another study [35], while *Ruthenibacterium lactatiformans*, *Flavonifractor plautii*, *Parabacteroides merdae*, *Ruminococcus torques*, and *Ruminococcus gnavus* were found to be less abundant. Increases in Firmicutes and *Lactobacillus* [34] have been reported in other research. It has been discovered that some MD components individually change the composition of the gut microbiota. For instance, eating walnuts was linked to higher relative abundances of the *Eubacterium eligens* group, *Leuconostocaceae*, *Lachnospiraceae*, and *Roseburia* [36]. The study found an inverse correlation between the lipid profile, blood pressure, and the relative abundance of Lachnospiraceae. A higher abundance of the Clostridiales vadin BB60 group was linked to the monounsaturated oleic acid [36]. Significant increases in *Bifidobacterium* [37], *Lactobacillus*, and *Roseburia* [38] have been reported in response to polyunsaturated fatty acids (PUFAs). These findings are summarized in Table 1.

In a different study, researchers discovered that people following the MD had higher *Prevotella*-to-*Bacteroides* ratios, suggesting that a diet rich in natural fiber and resistant starch has a good effect on patients’ bacterial composition [4]. A study that used a meal frequency questionnaire and a microbiota composition analysis was also carried out in a similar manner. Following completion, it was discovered that a greater F/B ratio was the result of poor diet adherence [2,39]. Individuals exhibited increased levels of SCFAs, *Bifidobacteria* counts, and Bacteroidetes when they adhered to the MD more closely [2].

Microbial metabolites provide some of the most compelling evidence for microbiota-mediated effects on human health. Tryptophan, an important amino acid included in many foods such as fish, chicken, dairy products, and grains [40], is one example of how it is metabolized by microbes in the MD. The microbiota have the ability to metabolize tryptophan into tiny compounds that can bind to the aryl hydrocarbon receptor (AhR) and cause enteroendocrine cells to secrete more glucagon-like peptide 1 (GLP-1) [40]. A decrease in AhR ligands and GLP-1 release, as well as compromised intestinal barrier function in animal models, have been linked to metabolic syndrome in both human and animal investigations [41]. The rise in microbiome gene representation linked to SCFA generation with the MD [35] is another example of the microbial-mediated influence of food; this is consistent with reported increases in fecal SCFAs [17].

A 12-month MD intervention was conducted in 612 non-frail or pre-frail subjects from five European countries (UK, France, The Netherlands, Italy, and Poland) as part of a study by Ghosh et al., which examined the effects of diet on inflammatory markers. There was a negative correlation found between the inflammatory markers of CPR, interleukin (IL)-17, and IL-2 and diet adherence. Positive levels of the anti-inflammatory cytokine IL-10 were also seen. Following the MD has been linked to several health benefits, such as an increased production of SCFAs and anti-inflammatory qualities that lower the risk of long-term inflammatory disorders like Type 2 diabetes mellitus (T2DM) [42].

#### 3.2.2. Western Diet

Reduced amounts of fruits and vegetables and high amounts of animal proteins, refined carbohydrates, and sugars—all heavy in calories—are characteristics of the WD [43] (Figure 1). Dietary fiber has a major influence on the microbiota, yet a normal WD is poor in it [44]. The bacterial diversity in the gut microbiome is impacted by a WD that increases *Bacteroides* spp., *Alistipes* spp., and *Bilophila* spp. while decreasing *Lactobacillus* spp., *Roseburia* spp., and *E. rectale*, the beneficial bacteria [2]. In a recent study, mice fed with a high-fat, high-sugar diet or a low-fat, high-sugar diet were used. The results showed that the mice fed with the high-fat, high-sugar diet had more Firmicutes and Mollicutes and fewer Bacteroidetes [45]. Researchers carried out an experiment on mice in a different study. The mice were randomly allocated to receive either a high-fat diet (HFD) or a regular chow diet. The researchers observed comparable changes in the composition of the gut microbiota. This confirmed earlier findings that the high-fat diet group included significant concentrations of Firmicutes and Proteobacteria. Furthermore, the HFD group also had greater levels of *Enterobacteriaceae*, *Escherichia*, *Klebsiella*, and *Shigella* according to this study. Overall, these studies’ findings indicate a relationship between alterations in the gut microbiota resulting from an HFD: a decrease in Bacteroidetes and an increase in Firmicutes [9]. A recent study by Suriyano et al. highlighted the significant impact of dietary macronutrients, specifically sugar and fat, on fecal bacterial counts and quantitative microbiome profiling in mice. The researchers found that fat, in particular, is a key factor driving changes in the gut microbiota composition. These alterations in gut bacteria are linked to the progression of obesity, diabetes, and local inflammation in various body tissues. The study emphasized that the type of macronutrients consumed can profoundly influence the gut microbiota, which play a critical role in the development of metabolic diseases and associated inflammatory conditions [46].

A WD is characterized by high quantities of additives produced from food processing, such as synthetic emulsifiers, and lower levels of natural foods containing dietary fiber. These emulsifiers have been demonstrated to modify the gut microbiota in mice, decrease the thickness of the mucus barrier, and encourage intestinal inflammation and metabolic disorders [47]. In a recent human randomized controlled clinical trial, microbial diversity, SCFA production, and free amino acid levels were observed to be reduced by dietary emulsifier supplementation during a brief dietary intervention in healthy participants. More specifically, there was an increase in *Lachnospiraceae* and *Roseburia* and a drop in *F. prausnitzii* and *Ruminococcus* levels. Lastly, some patients, but not all of them, experienced a decrease in their intestinal mucus thickness, even during a brief intervention [48].

High levels of animal-based proteins, which have the ability to alter the makeup and possible function of the gut microbiota, are another feature of a WD. Specifically, it has been observed that individuals following a WD had higher levels of trimethylamine N-oxide (TMAO) due to microbiota-dependent transformations converting l-carnitine and phosphatidylcholine, which are common in red meats [49]. Furthermore, high concentrations of ultra-processed foods and dangerous dietary additives, such as emulsifiers and artificial sweeteners, may contribute to the detrimental effects of the WD on the gut flora [50]. Junk foods that are characteristic of the WD are rich in artificial substances and preservatives, low in fiber, and can cause detrimental effects to the gut microbiota. These foods favor the proliferation of pathogenic bacteria and, acting at the same time, suppress beneficial bacteria, disrupting the microbiome’s balance and weakening the integrity of the intestinal barrier. The environment that highly processed meals create in the gut appears to alter the microbiota in a way that supports a variety of inflammatory diseases, such as metabolic disorders, IBD, and obesity [51].

Also, a WD is very limited in terms of the consumption of whole grain, which provides part of the daily needed dietary fiber and nutrients. Whole grains are essential contributors to a healthy and diverse gut microbiome because they act as prebiotics, stimulating the production of SCFAs beneficial for gut and general health. A lack of whole grains in the diet considerably reduces the number of good microbes and the production of SCFAs, which leads to inflammation. A randomized controlled trial by Vanegas et al. examined the effects of whole grain consumption on the gut microbiota in humans. Participants who consumed a diet rich in whole grains, such as oats and barley, showed a significant increase in the abundance of beneficial gut bacteria, particularly *Bifidobacterium* and *Lactobacillus*. In contrast, those who consumed refined grains exhibited a decrease in microbial diversity and a reduction in the production of short-chain fatty acids (SCFAs), which are crucial for maintaining gut health and reducing inflammation [52].

Additionally, a low consumption of fruits and vegetables is another significant factor. These foods are vital sources of vitamins, minerals, fiber, and phytochemicals that support gut health. Fruits and vegetables contribute to a balanced gut microbiome by fostering microbial diversity and inhibiting the growth of pathogenic bacteria. Their antioxidants and anti-inflammatory properties further help to protect the gut lining and promote overall digestive health. A study by Lakshmanan et al. explored the impact of fruit and vegetable intake on the gut microbiota in a cohort of healthy adults. The researchers found that fruits and vegetables can provide long-term health benefits by controlling the relative abundance of bacteria such as L-*Ruminococcus* and unclassified bacteria from the Erysipelotrichaceae family, possibly through a reduction in the pro-inflammatory response [53]. These findings are highlighted in Table 1.

#### 3.2.3. Vegetarian Diet

Vegetables, fruits, grains, legumes, and nuts make up the majority of a vegetarian diet, however, dairy and eggs are occasionally included [54]. Numerous food groups that are often included in a healthy vegetarian diet, such as those high in fiber, unsaturated fatty acids, and polyphenols, are also included in the MD. The saturated fatty acids obtained from animals are reduced as a result of not consuming red meat. A vegetarian diet has been linked to a decrease in the metabolites related to CVDs [49], such as acylcarnitine metabolites and l-carnitine [54]. These elements contribute to the explanation of some of the health advantages of a vegetarian diet, such as weight and cholesterol reductions [55]. A shift in the makeup of the gut microbiota, including a rise in alpha diversity, has also been linked to the vegetarian diet [56]. In particular, a vegetarian diet has been linked to higher relative abundances of the taxa that produce SCFAs, namely *Eubacterium biforme*, *F. prausnitzii*, *Eubacterium rectale* [56], and *Akkermansia* [54]. It has been shown that *Akkermansia* contributes to the preservation of the epithelial energy balance and integrity in the colons of animal models [57]. It is plausible that alterations in the microbiota linked to a vegetarian diet impact human health; however, more human data are required to substantiate a direct causal relationship.

According to the previous findings, high levels of *Prevotella* species have been associated with plant-based diets [58]. An animal study that used mice deficient in the miR-146a gene examined the effects of a plant-based diet rich in miR-146a on the microbial community. The study revealed that the microbiomes of the mice fed with the plant-based diet differed significantly from the microbiomes of the mice fed with the control diet, leading the researchers to conclude that a shift in microbial communities occurs when dietary fiber levels rise. For the mice consuming the plant-based diet, they discovered that *Bacteroides* and *Alloprevotella* significantly increased and Porphyromonadaceae and Erysipelotrichaceae decreased [59]. A human diet intervention study was carried out to investigate the bacterial composition based on what the participants indicated as their regular diet, in an effort to support the theory that a high-fiber diet alters the bacterial makeup. *Prevotella* enrichment was observed in ninety-eight vegetarian patients, but *Bacteroides* enrichment was observed in the microbiome environments of those who followed a conventional WD. The researchers also noticed that when 10 of the participants swapped diets, their microbiome compositions were altered within 24 h of the ingestion of the other diet [11]. Another related study produced comparable results. Additionally, they discovered that, in comparison to non-vegetarians, the vegetarian participants’ microbiomes were enhanced with *Prevotella* [60]. These key findings are highlighted in Table 1.

**Table 1:** Impact of dietary patterns on gut microbiota in relation to depression.

Dietary Pattern	Key Components	Impact on Gut Microbiome	Health Outcome	References
MD	- ↑ fruits, vegetables, whole grains, legumes, nuts, and olive oil - moderate consumption of fish and poultry - ↓ red meat and processed foods - ↑ fiber, polyphenols, and omega-3 fatty acids	- ↑ microbial diversity - ↑ growth of beneficial bacteria, e.g., *Bifidobacterium*, *Lactobacillus* - ↑ levels of *Faecalibacterium prausnitzii*, linked to anti-inflammatory effects - ↑ production of SCFAs	**Reduced risk of depression:** - ↑ gut health - ↑ neurotransmitter-modulating bacteria - ↓ inflammation - ↓ inflammatory markers - ↓ obesity and diabetes - gut–brain axis modulation	[2,33,34,36,39]
WD	- ↑ red and processed meats, refined grains, high-fat dairy, and sugars - ↑ processed foods, artificial additives - ↓ fiber, fruits, vegetables, and whole grains	- ↓ microbial diversity - ↑ growth of pro-inflammatory bacteria, e.g., *Proteobacteria*, *Bacteroides* - ↓ levels of SCFA-producing bacteria, e.g., *Roseburia*, *Faecalibacterium*	**Increased risk of depression:** - ↑ pro-inflammatory cytokines - gut–brain axis disruption - ↑ metabolic syndrome, obesity, and inflammation - ↑ prevalence of gut dysbiosis and leaky gut syndrome	[2,9,45,47,49,51]
Vegetarian	- ↑ fruits, vegetables, whole grains, legumes, nuts, and seeds - no meat; some variations allow dairy and eggs - rich in fiber, phytochemicals, and antioxidants - ↓ saturated fats and cholesterol	- ↑ microbial diversity route - ↑ growth of beneficial bacteria, e.g., *Bifidobacterium*, *Prevotella* - ↑ levels of SCFAs, particularly butyrate, linked to gut health - ↓ levels of bile-tolerant bacteria, which are linked to inflammatory responses	**Reduced risk of depression:** - improved gut health; reduced inflammation - lower risk of cardiovascular diseases, obesity, and type 2 diabetes - enhanced protection against certain cancers - lower inflammation and improved insulin sensitivity	[11,49,55,57,58]

↑, increase; ↓, decrease.

### 3.3. Impact of Macronutrients on Gut Microbiota Composition

#### 3.3.1. Carbohydrates

Food ingredients have a significant effect on the gut microbiota, affecting their composition in terms of richness and diversity. Consuming a lot of animal proteins, saturated fats, sugars, and salt may encourage the growth of pathogenic bacteria at the expense of beneficial bacteria, but eating complex polysaccharides and vegetable proteins may also increase the number of beneficial bacteria in the body [50]. Table 2 highlights the role of macronutrients in the gut flora.

Carbohydrates are primarily used by the body as a source of energy, and they significantly influence the composition and function of the gut microbiota. The gut microbiota, in turn, aid in the digestion of certain carbohydrates. The interaction between dietary carbohydrates and the gut microbiota plays a crucial role in shaping and altering the microbiota, affecting overall gut health and metabolic processes.

Dietary fiber is categorized as carbohydrates that are unable to be digested in the upper gastrointestinal system. They are not absorbed in the small intestine because they are not broken down by the host’s digestive system. The gut microbiota’s fermentation of carbohydrates is necessary for the breakdown of fibers. According to Rinninella et al., non-digestible oligosaccharides such as raffinose, stachyose, oligofructose, and inulin, resistant starches, and non-starch polysaccharides such as cellulose, hemicellulose, glucans, gums, and pectin make up fibers. Through saccharolytic fermentation, which is carried out by gut microbes in the colon, they produce monosaccharides, certain gases (methane and carbon dioxide), and SCFAs like butyrate, acetate, and propionate [61]. Dietary fiber consumption and the makeup of the gut bacteria influence the types and quantities of SCFAs that are present. Non-digestible complex carbohydrates have the ability to release particular microbial metabolites and encourage the growth of a wider range of microbial families and species with particular traits. Known also as microbiota-accessible carbohydrates (MACs), the primary sources of these carbohydrates include cereals, some fruits and vegetables, and human milk (human milk oligosaccharides) when a person is breastfeeding. Bananas, chicory root, onions, garlic, artichokes, asparagus, and other foods include MACs such as oligofructose and inulin [50]. Furthermore, dietary fiber possesses various physicochemical properties that significantly influence the composition and functionality of the gut microbiota. One key property is its solubility, which allows soluble fibers to form viscous gels in the gut, facilitating the fermentation process by beneficial bacteria, thus increasing the production of SCFAs [62].

There is growing interest in utilizing these carbohydrates as prebiotics to support gut health. Prebiotics promote the growth of beneficial bacteria such as *Lactobacillus* and *Faecalibacterium prausnitzii*. These bacteria metabolize prebiotics into SCFAs, which have various positive effects on gut health, including enhancing gut barrier function, reducing inflammation, and providing energy to colon cells. Consequently, these impacts enhance the function of the intestinal barrier, elevate insulin sensitivity, and improve the lipid profile [63]. According to Singh et al., inulin dose-dependently changed the makeup of the fecal microbiota in male rats fed with an HFD by suppressing the growth of Firmicutes phyla (such as *Roseburia* and *Clostridium* clusters I, IV, and XIV) and boosting the number of *Bifidobacterium* species and Bacteroidetes [64].

It has been demonstrated in animal models that the WD, which has a comparatively lower fiber content, decreases the variety of the gut microbiota and the quantity of *Bifidobacterium* [65]. Reducing the F/B ratio and raising the quantity of *Lactobacillus* sp. in rats fed with a high-fat, sucrose-enriched diet have been demonstrated to ameliorate gut dysbiosis [66]. Furthermore, MAC intake may be able to slow down the spread of the opportunistic diarrheal bacterium *Clostridium difficile*, according to a new mouse model [67]. Remarkably, a high-fat-MAC diet ameliorated cognitive deficits in a mouse model by influencing the gut microbiota–brain axis, which is triggered by high-fat food intake [68].

In order to disrupt their gut flora, humanized mice were given a high-fiber diet in a preclinical experiment before being given a low-quality feed. However, providing the studied animals with a plant polysaccharide-rich diet with a 15% weight-neutral detergent fiber content did not restore their microbial diversity and composition. Furthermore, it was noted that this disruption persisted for a number of generations [13]. Studies conducted in clinical trials have repeatedly shown that a high-fiber diet intervention increases the abundance of various beneficial bacteria in the feces, including *Lactobacillus* sp., *Prevotella* sp., and *Bifidobacterium* sp. [69]. In a randomized, double-blind clinical trial, galacto-oligosaccharides have been shown to significantly increase *Bifidobacterium* sp. and *Lactobacillus* sp. after four weeks of treatment [70].

#### 3.3.2. Fat

Microbial species modify the host’s lipid profile and obesity by metabolizing ingested fatty acids into different fatty acids [57]. Increased insulin resistance, intestinal permeability, and the inflammation of adipose tissue are the outcomes of consuming an HFD, particularly one high in saturated fat acids, which causes dysbiosis with a drop in Bacteroidetes and a rise in Firmicutes and Proteobacteria [71].

According to Wolters et al. there may be a positive correlation between the Enterobacteriaceae family, *Prevotella*, *Turicibacter*, and *Parabacteroides* genera and monounsaturated fatty acids (MUFAs) (palmitoleic, oleic, and eicosenoic) [72]. Conversely, in both healthy and ill models, including people at risk of metabolic syndrome, a diet high in MUFAs—rich in sesame, pumpkin seeds, rapeseed, extra virgin olive oil, and peanuts—showed favorable health effects with an enhanced variety of gut flora. Virgin coconut oil, human milk, and baby formulas all include medium-chain fatty acids (MCFAs), which may promote the growth of *Lactobacillus* and *Bifidobacterium* to improve metabolic and cognitive processes [73]. Medium-chain triglycerides (MCTs) enhance the gut microbial balance and gut barrier integrity, which, in turn, promotes energy expenditure, weight reduction, and lipid catabolism. However, diets high in coconut oil may raise the ratio of F/B, *Allobaculum*, *Clostridium*, *Lactobacillus*, and *Staphylococcus*, which may lead to metabolic problems and the inflammation of adipose tissue [61]. Since the human body is unable to produce PUFAs and must receive them from food, these fatty acids are referred to as “essential fatty acids”. PUFAs are mostly found in sunflower oil, nuts, seeds, and fatty fish. By raising the number of butyrate-producing Lachnospiraceae taxa and restoring the F/B ratio, omega-3 PUFAs can have a beneficial effect [61].

Research has shown that the amount and type of dietary fat significantly impact the composition of the intestinal microbiota [13]. Additionally, the abundance of sulfate-reducing bacteria (SRB) can disrupt the intestinal barrier by breaking down the disulfide bonds in mucus, causing defects and increasing intestinal inflammation [50]. A recent review demonstrated that MUFAs do not affect richness or diversity indices, the distribution of phyla, or the ratio between Bacteroidetes and Firmicutes [8]. However, at the family and genus levels, diets high in monounsaturated fatty acids, such as those found in the MD, may positively correlate with the genera *Parabacteroides*, *Prevotella*, and *Turicibacter*, as well as the family Enterobacteriaceae. Conversely, these diets may negatively correlate with the genus *Bifidobacterium*.

It has been repeatedly demonstrated that a diet high in saturated and/or total fat negatively impacts the gut microbiota. Diets high in total and saturated fats have been found to have a detrimental impact on the richness and diversity of the gut microbiota, according to fifteen clinical reports (six of which were randomized controlled interventional trials and nine of which were observational studies) [72]. Carefully monitored feeding trials in mice confirmed these results, demonstrating that diets with fat ranging from 44% to 72% raised the gut microbiota’s F/B ratio [74]. In a recent controlled-feeding clinical trial, 40% fat consumption by young adults in good health was found to be linked to unfavorable changes in the gut microbiota. Specifically, the intervention led to a decrease in the abundance of beneficial *Fecalibacterium* bacteria and an increase in harmful *Bacteroides* and Alistipes species, which are known to be prevalent in patients with T2DM. On the other hand, 20% fat consumption increased the amount of *Fecalibacterium* and *Blautia* spp. in the gut microbiota [75].

According to Yang et al., unsaturated fat enhances the abundance of *Akkermansia* and *Bifidobacterium* and decreases harmful bacteria like *Streptococcus* and *Escherichia* sp. Conversely, saturated fat consistently lowers health-beneficial microbes like *Bifidobacterium* and *Fecalibacterium*. Furthermore, depending on the fat quality, saturated and unsaturated fats might have different effects on human health. Saturated fat can raise the F/B ratio, whereas unsaturated fat can lower it [13].

#### 3.3.3. Proteins

Proteins are linear chains of amino acids joined by peptide bonds. In the distal colon, the primary microbial phyla, Firmicutes, Bacteroidetes, and Proteobacteria, ferment amino acids. Proteolytic fermentation has the ability to produce branched-chain fatty acids like isobutyrate, 2-methyl butyrate, and isovalerate, as well as potentially toxic substrates like ammonia, nitrosamines, and TMAO in smaller amounts than saccharolytic fermentation [61]. The amount and quality of the dietary proteins that the gut microbiota digest, especially those with plant or animal origins, determine the balance of the gut microbiota populations and the generation of these metabolites. An increased amount of bile-tolerant anaerobic bacteria, such as *Bacteroides*, *Alistipes*, and *Bilophila*, may result from a diet high in animal proteins, such as red meat and dairy products. This could increase the amount of TMAO, a substance with proatherogenic potential that may contribute to CVD. More specifically, animal diets like red meat include large amounts of the amino acid L-carnitine, which may be a key factor in the elevated risk of CVD [76,77].

A traditional WD that includes a high amount of animal-based proteins may promote the growth of SRB, such as *Desulfovibrio* spp., which produces hydrogen sulfide from dietary inorganic sulfur and sulfated amino acids, such as taurine, methionine, and cysteine. This could lead to an increase in gut inflammation [78]. Conversely, consuming plant-based proteins like glycated pea proteins may lead to a rise in the number of good bacteria like *Lactobacillus* and *Bifidobacterium* and a drop in the number of *Bacteroides fragilis* and *Clostridium perfringens* [79]. Pulses, which mostly consist of lentils, beans, chickpeas, and peas, have garnered more interest in recent years due to their potential as a sustainable alternative to animal proteins. In fact, eating pulses has been linked to favorable changes in the gut flora in both humans and animals, including enhanced development of the butyrate- and acetate-producing species *Bifidobacterium*, *Faecalibacterium*, *Clostridium*, *Eubacterium*, and *Roseburia* [80]. To cut down on gut-inflammation-related proteins like cysteine and methionine, a diet high in plant-based proteins, such as pulses, is an excellent substitute for animal proteins. Moreover, they are rich in bioactive substances and resistant starches, both of which have a well-established beneficial effect on the gut microbial balance [81].

Data from animal models indicate that the makeup of the gut microbiota is influenced by the quality of the proteins. For instance, a preclinical study [82] revealed that cheese whey proteins, as opposed to casein, might function as growth factors for the fecal counts of *Lactobacilli* and *Bifidobacteria*. Mice with a HFD-induced F/B ratio were demonstrated to respond differently to mung bean protein. In a model of HFD mice, the mung bean protein also boosted the abundance of the Ruminococcacea family. The authors postulated that the Ruminococcacea family members’ bile acid (BA) metabolism would have benefited the health of the HFD mice in light of this result [83].

### 3.4. Impact of Micronutrients and Phytochemicals on Gut Microbiota Composition

#### 3.4.1. Vitamins

Vitamins are organic compounds that are essential in very small amounts for supporting normal physiological function. They frequently perform a number of functions in the body, the most significant of which is acting as cofactors for enzymes. Since our bodies cannot produce enough of certain vitamins to satisfy our daily needs, the diet is the main source of these nutrients. However, other vitamins, such as vitamin K and B group vitamins, are produced by the gut bacteria. They are necessary for immune system function, cell development and differentiation, and energy metabolism control. The gut microbiota are capable of producing thiamine, riboflavin, niacin, biotin, pantothenic acid, folate, and vitamin K, among other vitamins. Moreover, a number of studies have demonstrated that vitamin D may influence the microbiota’s makeup, altering it and raising the number of potentially advantageous bacterial strains [61]. The gut microbiota may be influenced by antioxidants like carotenoids. According to recent research, lutein derived from black currants has been proven to both decrease Bacteroidetes and *Clostridium* spp. and increase bifidobacteria and lactobacilli. However, the gut microbiota mediate the anti-inflammatory benefits of beta-carotene [8].

#### 3.4.2. Minerals

Essential micronutrients such as minerals and trace elements are involved in human metabolism and actively interact with the gut flora [84]. Human diseases can result from both excesses and shortages of certain micronutrients. For example, a lean phenotype has been linked to alterations in the gut microbiota that have been linked to high calcium consumption [85]. In a human trial, an eight-week course of 1000 mg of calcium per day increased the amount of *Clostridium* XVIII in men’s fecal samples [86].

The effects of iron supplementation, which is frequently used to treat iron insufficiency, on the gut flora have been inconsistent. However, following iron consumption, several studies have consistently shown increases in harmful bacteria and decreases in good bacteria [87]. According to research on animals, excessive iron can lead to intestinal dysbiosis, which can decrease the number of some Lachnospiraceae family members and the genus *Allobaculum*, while increasing the number of bacteria from the Defluviitaleaceae, Ruminococcaceae, and Coprococcus families [88]. These results have been corroborated by in vitro investigations, which have demonstrated that elevated iron concentrations can raise harmful metabolites, reduce commensal bacteria, and boost pathogenic bacteria’s pathogenicity [89].

Another important element that preserves the epithelial integrity is zinc, which may act by influencing the good gut microbiota [90]. According to Yang et al., grill chickens with a chronic zinc shortage experienced a considerable rise in Proteobacteria abundance and a decrease in Firmicutes. There are, however, insufficient clinical studies on the effects of dietary zinc on the human gut flora [13]. The impact of iodine supplementation on the gut flora seems to be influenced by the amount of fat in the diet. Iodine corrected the thyroid hormone status in an HFD animal model, but it also led to gut dysbiosis, which was characterized by a decrease in helpful bacteria like *Fecalibacterium prausnitzii* and an increase in dangerous bacteria [91]. On the other hand, the same iodine dose boosted good bacteria such as *Bifidobacterium*, *Lactobacillus*, *Fecalibacterium*, and *Allobaculum* in the setting of a low-fat diet (LFD) [91].

In conclusion, research on the effects of micronutrient shortage or supplementation is the main focus of current studies, even though there is little information on the precise processes by which minerals and trace elements affect the gut microbiota. It has been demonstrated that in experimental animals, calcium influences the gut microbiota, including *Ruminococcacea*, *Bifidobacterium*, and *Akkermansia*, as well as the ratio of Bacteroidetes to *Prevotella*. It is unknown how magnesium affects the gut microbiome, although some research has indicated that a sudden lack of the mineral may alter the balance of good gut bacteria. While some clinical trials have found no impact, iron supplementation generally increases harmful bacteria and lowers helpful bacteria, such as *Bifidobacterium*, in babies. Iron administration routes and chemical forms seem to have important effects. Table 2 highlights the roles of micronutrients in the gut flora. There is a clear need for more comprehensive preclinical and human intervention studies to fully understand the role of specific minerals and trace elements in modulating the gut microbiota [13].

**Table 2:** Impact of dietary nutrients on gut microbiota.

Nutrients	Dose and Treatment Duration/Test Model	Potentially Beneficial Microbiota	Potentially Detrimental Microbiota	Reference
Dietary carbohydrates	Model: almond-based low carbohydrate diet as reference/clinical trials (45 T2DM patients) Duration: 3 months Age: >18 years Location: Hospital of Soochow University, Suzhou, China	↑ *Roseburia* sp. ↑ *Ruminococcus* ↑ *Eubacterium*		[92]
	Model: oligofructose-enriched inulin (Synergy 1) (10 g/day), n = 18 and placebo (maltodextrin; 7 g/day), n = 16 as reference/clinical trials (34 pediatric celiac disease patients, 62% females, on a gluten-free diet) Duration: 3 months Age: mean age 10 years Location: University of Warmia and Mazury, Olsztyn, Poland	↑ *Bifidobacterium* sp.		[93]
Dietary fat	Model: HFD (60% fat) and baseline as reference/mice model, n = 8 Duration: 14 weeks Age: 4 to 5 weeks Location: Jiangnan University, Wuxi, China	↓ *Bifidobacterium* sp. ↓ *Lactobacillus* sp. ↓ *Faecalibaculum*, sp. ↓ *Akkermansia*. sp.	↑ *Desulfovibrionaceae* sp. ↑ *Mucispirillum* sp.	[94]
	Model: fat diet (20%) and the baseline as reference/clinical trial (52% women) n = 217 Duration: 6 months Age: 18 to 35 years Location: Army General Hospital, north China and Zhejiang University, south China	↑ *Fecalibacterium* sp. ↑ *Parabacteroides* sp.		[75]
	Model: moderate-fat diet (30%) and the baseline as reference/clinical trial (52% women) n = 217 Duration: 6 months Age: 18 to 35 years Location: Army General Hospital, north China and Zhejiang University, south China	↓ F/B		[75]
	Model: HFD (40%) and the baseline as reference/clinical trial (52% women) n = 217 Duration: 6 months Age: 18 to 35 years Location: Army General Hospital, north China and Zhejiang University, south China	↓ F/B ↓ *Fecalibacterium* sp.	↑ *Alistipes* sp. ↑ *Bacteroides* sp.	[75]
Dietary protein	Model: high-animal-protein-based diet (514 g/kg) and baseline as reference/mice model Duration: 3 weeks Age: shortly after weaning Location: Czech academy of sciences, Prague		↑ *Escherichia* sp. ↑ *Staphylococcus* sp. ↑ *Enterococcus* sp.	[95]
	Model: plant-protein-based control diet (176 g/kg) and baseline as reference/mice model Duration: 3 weeks Age: shortly after weaning Location: Czech academy of sciences, Prague		↑ *Enterococcus* sp.	[95]
	Model: C57BL/6 DSS-treated mice with isocaloric diets with 53% protein and the diets with 30% protein as reference/DDS-treated mice model, n = 132 Duration: 3 days Age: 7 weeks Location: Université Paris-Saclay, France	↑ *Desulfovibrio* sp. ↑ *Bacteroides* sp.	↑ *Alloprevotella* sp. ↑ *Haemophilus* sp. ↑ *Klebsiella* sp.	[96]
Minerals	Model: supplementation of 1000 mg calcium + 1000 mg phosphorus/day and the supplementation of 1000 mg phosphorus/day as reference/clinical trials (men n = 30, women n = 32), Duration: 8 weeks Age: 29 ± 7 years Location: University Jena, Germany	↑ *Clostridium* sp.		[97]
	Model: high-iron-fortified formula (6.4 mg Fe/day) and iron drops (no-added-iron formula with liquid ferrous sulfate supplementation (5.7 mg Fe/day) as reference/clinical trials, n = 53 Duration: 45 days Age: 6 months Location: Sweden	↑ *Lactobacillus* sp.	↓ *Ruminococcus* sp.	[98]
	Model: 18 μg/day iodine and control group as reference/mice model, n = 24 Duration: 8 weeks Age: 3 weeks Location: Ningbo University, China	↑ *Bifidobacterium* sp. ↑ *Lactobacillus* sp. ↑ *Fecalibacterium* sp. ↑ *Allobaculum* sp. ↑ *Roseburia* sp.		[91]
Vitamins	Model: one dose of 50,000 IU vitamin A and placebo as reference/clinical trial, Duration: 15 weeks Age: 48 h of birth for IU treatment, placebo–early (6–15 week) or late (2 year) infancy Location: Dhaka, Bangladesh	↑ *Bifidobacterium* sp. ↑ *Akkermansia* sp.		[99]
	Model: a dose of 40,000 IU vitamin D once weekly using two capsules of 20,000 IU (Plenachol, Encap) and baseline as reference/clinical trial (patients with vitamin D deficiency: 25[OH] vitamin D < 50 nmol/L) Duration: 1 month Age: NA Location: St Mark’s Hospital, London, UK	↑ *Enterobacteria* sp.		[100]
Polyphenols	Model: HFD with blackberry anthocyanin rich extract (25 mg/kg body weight per day) and HFD as reference/rat model n = 6 Duration: 17 weeks Age: NA Location: NA	↑ *Akkermansia* sp.	↓ *Ruminococcus* sp.	[101]
	Model: tart cherry juice consumption (8 oz/day) and baseline as reference/clinical trials, n = 10 Duration: 5 days Age: 23–30 years Location: NA	↑ *Bifidobacterium* sp. ↑ *Prevotella* sp. ↑ *Bacteroides* sp.	↓ *Ruminococcus* sp.	[102]
	Model: different concentrations of grape phenolic compounds (2.5 and 5 mg/kg/day diluted in 0.1% Dimethyl Sulfoxide) and the control group (0.1% Dimethyl Sulfoxide alone) as reference/rat, n = 6 Duration: 2 months Age: NA Location: NA	↑ *Bifidobacterium* sp.		[103]

↑, increase; ↓, decrease; NA, not applicable.

### 3.5. Impact of Specific Dietary Components on Gut Microbiota Composition

#### 3.5.1. Prebiotics

Prebiotics have been recognized as dietary components that host beneficial bacteria preferentially used to give a health advantage [104]. The majority of prebiotic substances are naturally occurring carbohydrates with a range of molecular structures found in food. Prebiotics include lactulose, inulin, galactooligosaccharides, and fructooligosaccharides. They are present in a variety of foods, including bananas, onions, garlic, and whole grains. In addition to carbohydrates, other compounds that may have prebiotic effects include polyphenols and PUFAs [8].

Prebiotics mainly function by giving beneficial microorganisms in the stomach a source of food. Since human enzymes cannot break them down, they pass through to the colon undigested, where the gut flora ferment them [105]. Some microorganisms, like those in the genus Bacteroides, can use high-molecular-weight polysaccharides, but others, like bifidobacteria, can metabolize low-molecular-weight carbohydrates very effectively, because they have a variety of cellular and extracellular glycosidases and specific transport systems [8]. During this fermentation process, SCFAs such as butyrate, propionate, and acetate are produced [106]. Prebiotics aid in maintaining a healthy gut microbiota composition by reducing the number of harmful bacteria and specifically promoting the development and activity of good gut bacteria like *Lactobacilli* and *Bifidobacteria*. Concurrently generated metabolites can boost gut barrier function, control the immune system, supply energy to the colon’s lining cells, and even affect brain function via the gut–brain axis [107]. For instance, oligofructose increases the expression of many genes involved in mucus formation, glycosylation, and secretion, the expression of both transmembrane and secreted mucins, and the differentiation and quantity of goblet cells, preventing HFD-induced obesity in mice. These findings were linked to notable alterations in the composition of the gut microbiota, with oligofructose considerably raising the abundance of the bacterial taxa *Ruminococcaceae*, *Odoribacter*, *Akkermansia*, and two undiscovered *Muribaculaceae*. It is interesting to note that all of these bacterial species had a positive correlation with mucus layer indicators and a negative correlation with metabolic parameters [108].

#### 3.5.2. Probiotics

Probiotics were defined as “live microorganisms which, when administered in adequate amounts, confer a health benefit on the host” by the Food and Agriculture Organization of the United Nations and the World Health Organization in 2001. This definition is currently the most widely accepted [105]. Probiotics are diverse organisms derived from many families of bacteria and yeasts. Along with the yeast *Saccharomyces cerevisiae*, some of the most well-known species of bacteria utilized as probiotics include *Lactobacillus*, *Bifidobacterium*, *Enterococcus*, and *Streptococcus*. There are several species in each genus, and there are numerous strains within each species [105]. Probiotics are typically thought to provide strain-specific health benefits. Probiotics work through a variety of intricate processes that vary according to the strain. Nevertheless, a few shared mechanisms have been identified. Probiotics can change the makeup of the gut microbiota, improve the operation of the intestinal barrier, modify the immune system, and compete with pathogens for nutrients and binding sites on the intestinal wall [8].

Additionally, they have the ability to produce metabolites and antimicrobial compounds that may have an impact on host health either directly or indirectly. By interacting through the gut–brain axis, they can affect the host’s neurological system [109]. Probiotics have the previously mentioned capacity to alter the makeup and/or activity of the gut microbiota, which may aid in the prevention or treatment of a number of illnesses, including metabolic syndrome, irritable bowel syndrome (IBS), and IBD [105].

#### 3.5.3. Polyphenols

Polyphenols include flavonoids, phenolic acids, stilbenes, and lignans from fruits, vegetables, cereals, tea, coffee, and wine [110]. Polyphenols are being increasingly recognized for their role in preventing diseases such as diabetes and obesity, attracting significant scientific interest. However, their absorption and bioavailability in humans are still subjects of debate. Researchers generally agree that the interactions between the intestinal microbiota and phenolic compounds significantly impact the bioavailability of these compounds [61]. Studies have shown that phenolic compounds can promote the growth of beneficial microorganisms; for instance, anthocyanins have been found to increase the populations of *Bifidobacterium* spp., *Lactobacillus*, and *Enterococcus* spp. Additionally, the microbiota are crucial in modulating the transformation of phenolic compounds into smaller metabolites, thereby influencing the bioavailability and beneficial properties of proanthocyanidins [111].

According to research conducted in vitro, polyphenols may influence the composition of the human gut microbiota by suppressing the growth of potentially harmful bacteria (like *Helicobacter pylori* and *Staphylococcus* sp.) and promoting the development of potentially helpful bacteria (like *Lactobacillus* and *Bifidobacteria*) [112]. According to Wang et al., polyphenols have been shown in animal studies to alter gut microorganisms, microbial diversity, and F/B ratio. This and other research has indicated that the primary mechanism underlying the health advantages of polyphenols in humans is their prebiotic-like properties [113].

#### 3.5.4. Resistant Starch

Resistant starch is a type of carbohydrate that resists digestion in the small intestine and ferments in the large intestine, acting as a prebiotic to beneficial gut bacteria [114]. This fermentation process produces SCFAs, such as butyrate, which have significant health benefits, including anti-inflammatory properties and improved gut barrier function. Studies have shown that diets high in resistant starch can positively alter the gut microbiota composition by increasing the abundance of beneficial bacteria like *Bifidobacterium* and *Lactobacillus*. Additionally, resistant starch has been linked to improved metabolic health markers, such as a better insulin sensitivity and a reduced risk of colorectal cancer. These findings underscore the importance of incorporating resistant-starch-rich foods, such as legumes, unripe bananas, and cooked and cooled potatoes, into the diet to support gut health and reduce inflammation [106].

## 4. Gut Microbiota and Inflammation

### 4.1. Mechanisms of Gut Microbiota–Immune System Interaction

The diverse microbial community, referred to as the gut microbiota, inhabits the gastrointestinal tract and engages in a dynamic and reciprocal relationship with the host immune system. This crosstalk between the gut microbiota and the immune system is fundamental for the development and function of the immune system, as well as for the maintenance of gut homeostasis and overall health.

From birth, the gut microbiota are instrumental in shaping the host immune system. Early colonization by beneficial microbes influences the maturation of immune cells, such as T cells, and the development of gut-associated lymphoid tissue. These interactions help to train the immune system to distinguish between harmful pathogens and benign antigens, preventing overreactions that could lead to allergies or autoimmune diseases. Studies have shown that germ-free animals, which lack gut microbiota, have underdeveloped immune systems and are more susceptible to infections and immune-mediated disorders [115].

The gut microbiota influence the immune system’s regulatory mechanisms, promoting anti-inflammatory responses and maintaining immune tolerance. Certain gut bacteria, such as *Bifidobacterium* and *Lactobacillus*, induce the production of anti-inflammatory cytokines like IL-10 and regulatory T cells (Tregs), which help to suppress excessive immune responses and maintain intestinal homeostasis [115]. Dysbiosis, or an imbalance in the gut microbiota, can disrupt these regulatory pathways, leading to chronic inflammation and contributing to various inflammatory diseases, including IBD, rheumatoid arthritis (RA), and metabolic syndrome [116].

### 4.2. Gut Barrier Function and Permeability

The intestinal barrier is a dynamic structure that may react to a variety of stimuli and interface with them. It is made up of several components. It plays a crucial role in maintaining homeostasis by allowing the absorption of nutrients and water while preventing the entry of harmful pathogens, toxins, and antigens [117]. The gut microbiota contribute to the integrity of the gut barrier, a crucial line of defense against pathogens [118]. Beneficial bacteria produce SCFAs like butyrate, which nourish colonocytes and strengthen the tight junctions between epithelial cells. This prevents the translocation of harmful bacteria and their toxins into the bloodstream, reducing the risk of systemic inflammation and infection. Additionally, the gut microbiota compete with pathogenic bacteria for nutrients and attachment sites, further protecting the host from infections [119].

The gut barrier comprises several layers that work together to maintain its integrity and function. The first line of defense is the mucus layer, which covers the epithelial cells lining the gut. This layer is composed of mucins and glycoproteins that are secreted by goblet cells [118]. The mucus traps pathogens and particles, preventing them from reaching the epithelial surface. It also provides a habitat for commensal bacteria, which play a role in barrier function and immune modulation [120]. Though mucus protects epithelial cells as the first line of defense and is composed of the same biological elements throughout the gastrointestinal tract, its characteristics fluctuate depending on the region. The mucus layers in the small and large intestines are not the same thickness. The small intestine’s primary roles include the digestion of food and nutrient absorption; in addition, it receives far less exposure to the microbiota than the colon. Thus, it contains a single layer. Conversely, in the large intestine, the quantity and kind of bacteria that reside there dictate the thickness of the mucus layer. The mucus is arranged into two layers: the loose outer layer and the stiff inner layer. Though there are notable morphological distinctions between these two levels, their peptide contents are nearly identical. In a steady state, the inner mucus layer is free of germs, because it is highly organized into a flat, lamellar structure and stays fixed to the epithelial cells. It also prevents bacteria from penetrating. The inner layer and the outer layer are separated by the relative demarcation line [118]. The middle epithelial cell layer is the primary physical barrier, consisting of a single layer of enterocytes, goblet cells, enteroendocrine cells, and paneth cells. These cells are interconnected by tight junctions, adherens junctions, and desmosomes, which regulate the permeability of the barrier. Tight junctions are dynamic structures composed of proteins such as claudins, occludins, and zonula occludens, which can open or close in response to various stimuli, thus controlling the paracellular transport of substances [7]. Underlying the epithelial layer is the lamina propria, which contains immune cells such as macrophages, dendritic cells, and lymphocytes, as well as structures like Peyer’s patches. These immune cells constantly monitor and respond to microbial and antigenic stimuli, playing a crucial role in maintaining the integrity of the gut barrier and preventing systemic infections [121]. The gut microbiota, composed of trillions of microorganisms, form a complex and dynamic community that interacts with the gut barrier. Beneficial microbes help to maintain this barrier by producing metabolites such as SCFAs, which enhance the function of epithelial cells and tight junctions. Dysbiosis, or an imbalance in the gut microbiota, can lead to barrier dysfunction and increased permeability [120].

The integrity and function of the gut barrier are regulated by various factors, including the diet, microbial metabolites, immune responses, and genetic predispositions. Dietary components can significantly influence gut barrier function. Fiber-rich diets promote the production of SCFAs by the gut microbiota, which strengthens the epithelial barrier and modulates immune responses. Conversely, diets high in fat and sugar, typical of the WD, can disrupt the gut barrier by promoting inflammation and altering the composition of the microbiota [122]. SCFAs, such as butyrate, propionate, and acetate, produced by the fermentation of dietary fibers, play a crucial role in maintaining gut barrier integrity. Butyrate, in particular, is a primary energy source for colonocytes and enhances the expression of tight junction proteins, thereby reducing permeability. Other microbial metabolites, such as indole derivatives and secondary BAs, also contribute to barrier function [6]. Immune cells in the gut, including regulatory T cells (Tregs) and innate lymphoid cells, produce cytokines and other signaling molecules that modulate barrier function. Anti-inflammatory cytokines, such as IL-10, help to maintain barrier integrity, while pro-inflammatory cytokines, such as tumor necrosis factor-alpha (TNF-α), can disrupt tight junctions and increase permeability [123]. Genetic predispositions can affect the structure and function of the gut barrier. Mutations in genes encoding for tight junction proteins, mucins, and other components of the epithelial layer can lead to barrier dysfunction and increased susceptibility to diseases like IBD and celiac disease [7].

**Figure 2:**
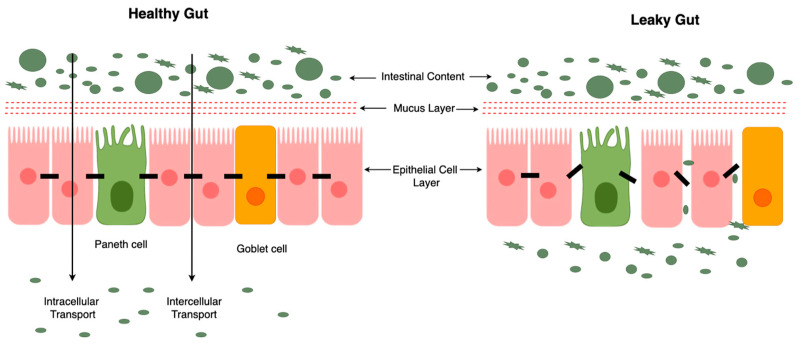
Gut barrier function and permeability (leaky gut situation). In a healthy gut, intestinal contents selectively pass through epithelial cells into the bloodstream, primarily via transcellular transport. This process is tightly regulated by tight junctions between epithelial cells, which restrict the passage of unwanted substances. In a leaky gut, the integrity of the intestinal barrier is compromised due to a reduced thickness of the mucus layer and the loosening of tight junctions. This compromised barrier allows pathogens and toxins to cross the intestinal lining and enter the bloodstream.

Dysfunction of the gut barrier, often referred to as “leaky gut” (Figure 2) is characterized by increased intestinal permeability, allowing for the translocation of pathogens, toxins, and antigens into the bloodstream. This can trigger systemic inflammation and contribute to the development of various diseases. Increased gut permeability is a hallmark of IBD, including Crohn’s disease (CD) and ulcerative colitis (UC). Dysbiosis and inflammation disrupt the barrier, leading to chronic intestinal inflammation [124]. Gut barrier dysfunction is associated with metabolic disorders such as obesity, T2DM, and non-alcoholic fatty liver disease. Increased permeability allows for the translocation of endotoxins, such as lipopolysaccharides (LPS), which induce systemic inflammation and insulin resistance [125]. Leaky gut has been implicated in the pathogenesis of autoimmune diseases, including celiac disease, RA, and T1DM. Increased permeability allows for the passage of antigens that trigger autoimmune responses [126].

A significant contributing factor to leaky gut is a reduction in the thickness of the intestinal mucus layer. This mucus layer, secreted by goblet cells, acts as a protective barrier between the gut lumen and the epithelial cells. When the mucus layer becomes thinner, it provides less protection, allowing for pathogens and toxins to come into closer contact with the gut lining. Additionally, a decrease in the number of goblet cells further exacerbates this problem by reducing the production of mucus. This reduction compromises the gut’s ability to maintain a robust barrier and can lead to increased intestinal permeability [127].

Furthermore, leaky gut is associated with changes in the microbial composition and function. A compromised mucus layer and fewer goblet cells disrupt the normal habitat for beneficial gut bacteria, leading to an imbalance in the microbiota. This imbalance can contribute to systemic inflammation and the development of various health issues, including inflammatory bowel disease, metabolic disorders, and even neuropsychiatric conditions. Therefore, maintaining the integrity of the intestinal mucus layer and ensuring adequate goblet cell function are essential for preserving the gut barrier function and overall health [127,128].

Emerging evidence suggests a link between gut barrier dysfunction and neurological disorders, such as autism spectrum disorder and depression. The gut–brain axis, a bidirectional communication system between the gut and the brain, is influenced by gut permeability and microbial metabolites [129]. The gut barrier is a complex and dynamic structure essential for maintaining intestinal and systemic health. Its integrity and function are regulated by various factors, including diet, microbial metabolites, immune responses, and genetic predispositions. Understanding the mechanisms underlying the gut barrier function and permeability is crucial for developing strategies to prevent and treat diseases associated with barrier dysfunction.

### 4.3. Production of Metabolites and Their Effects on Inflammation

The gut microbiota play a crucial role in the metabolism of dietary components, leading to the production of various metabolites that significantly influence host health. These metabolites include SCFAs, BAs, vitamins, amino acid derivatives, and other bioactive compounds. The production of these metabolites is a result of the fermentation and biotransformation processes carried out by the gut microbiota, and they can have profound effects on the host’s physiology and immune responses.

#### 4.3.1. Short-Chain Fatty Acids

In the large intestine, certain bacterial species digest carbohydrates to form short aliphatic tails of six carbons, which are called SCFAs. These SCFAs are produced under anaerobic conditions [130]. The main source of SCFAs, including acetate (C2), propionate (C3), and butyrate (C4) (Table 3), is dietary fibers, generally referred to as prebiotics. However, other nutrients, such as proteins and peptides, can also be converted into SCFAs. Bacteria digest oligofructose, arabinoxylan, inulin, and pectin to produce acetate, propionate, and butyrate in the proximal colon at considerable levels (70–140 mM) and at lesser amounts (20–70 mM) in the distal colon and distal ileum (20–40 mM) [131]. However, these proportions may differ based on variables such as the host genotype, fermentation location, microbiota makeup, and diet [132].

The portal vein delivers acetate and propionate to the liver, whereas the colonocytes mainly use butyrate. Propionate either remains in the liver or is discharged systemically into the peripheral venous system after being broken down by hepatocytes. As a result, often, only acetate is found in the peripheral blood [131]. Previous studies have shown that particular species with specific enzymes could be responsible for producing the different SCFAs. Microorganisms may also synthesize mono- and disaccharides from dietary fiber and other carbohydrates due to these enzymes. These saccharides are used by microbes to produce SCFAs [133].

The primary method by which enteric and acetogenic bacteria produce acetate is called reductive acetogenesis. As an acetogenesis process, the oxygen-sensitive Wood–Ljungdahl path is thought to be the most effective way for bacteria to generate acetate [131]. Bacteria metabolize carbohydrates in a few different ways to produce propionate. These routes consist of propanediol, acrylate, and succinate. Certain species of Firmicutes and Bacteroidetes favor the succinate route [134]. The synthesis of butyrate can proceed via two routes: butyrate synthesis from acetoacetyl-CoA, which is produced when two acetyl-CoA molecules combine. Butyryl-CoA: butyrate is produced by acetate CoA-transferase from butyryl-CoA. Butyryl-CoA: acetyl-CoA is extended to form butyrate by the enzyme acetate CoA-transferase, which is present in *Eubacterium*, *Roseburia*, *Anaerostipes*, and *Faecalibacterium prausnitzii* [135,136]. Butyrate kinase and phosphotransbutyrylase provide an additional pathway. For example, butyrate kinase is required by certain *Clostridium* species and several *Coprococcus* species in the Firmicutes family to produce butyrate [137] (Table 3).

In the organism, SCFAs are involved in a variety of physiological and immunological functions. SCFAs exert multiple anti-inflammatory effects through various mechanisms: 1. the regulation of immune cells, 2. the maintenance of gut barrier integrity, 3. epigenetic modulation, and 4. the modulation of metabolic pathways.

SCFAs influence the differentiation and function of immune cells, including T cells, macrophages, and dendritic cells. Butyrate and propionate enhance the differentiation of regulatory T cells (Tregs), which play a crucial role in maintaining immune tolerance and suppressing excessive inflammatory responses. SCFAs also inhibit the activation of pro-inflammatory macrophages and dendritic cells, reducing the production of pro-inflammatory cytokines such as IL-6 and TNF-α [138]. According to research by Souders et al., butyrate prevented TNF-α-induced reductions in Matrix metalloproteinase and mitochondrial-to-intracellular calcium ratios. This implies that butyrate may protect colonocytes from TNF-α-induced cytotoxicity by reducing the flow of calcium through the mitochondria [139].

Butyrate is particularly important for maintaining the integrity of the gut epithelial barrier. It provides energy to colonocytes and promotes the expression of tight junction proteins, which enhance the barrier function and prevent the translocation of pathogens and their toxins into the bloodstream. By maintaining the gut barrier integrity, SCFAs reduce systemic inflammation and protect against conditions like “leaky gut” syndrome [140]. SCFAs affect the host’s enterocytes and digestive function locally. For example, at least 60–70% of the energy needed for colonic differentiation and proliferation is provided by butyrate, a key metabolic substrate for colonocytes [141].

In addition to supplying colonocytes’ energy, SCFAs in the gut affect the colonic blood flow, colonic motility, and pH of the gastrointestinal environment, all of which have an impact on the absorption of nutrients and electrolytes. These effects may be mediated via the activation of G protein-coupled receptors, such as GPR41, GPR43, and GPR109A [142,143]. The activation of these receptors by SCFAs can lead to anti-inflammatory signaling pathways, further reducing inflammation and promoting metabolic health [144].

SCFAs, especially butyrate, act as histone deacetylase inhibitors, leading to the hyperacetylation of histones and changes in gene expression. This epigenetic modulation can suppress the expression of genes involved in inflammation and immune responses, contributing to the anti-inflammatory effects of SCFAs [145].

However, butyrate causes human monocytes to produce more IL-10 and less IL-12, which, in turn, prevents the synthesis of proinflammatory molecules, including nitric oxide, TNF-α, IL-1b, and nuclear factor kappa B (NF-kB) activity. Moreover, butyrate activates caspases 8 and 9, which, in turn, causes neutrophil death and inhibits the high mobility group box-1 [146]. In human goblet-like cells, line LS174T and T84 epithelial cells, butyrate, and propionate have been shown to induce the production and secretion of Mucin 2. It appears from this that SCFAs are essential bacterial compounds for maintaining gut integrity [147].

**Table 3:** SCFA production by microbes in the gut.

SCFA	Receptor	Pathway/Reaction	Producers	References
Acetate	GPR43	Via acetyl-CoA	*Akkermansia municuphila* *Bacteroides* spp. *Bifidobacterium* spp. *Prevotella* spp. *Ruminococcus* spp.	[148]
		Wood-Ljungdahl pathway	*Blautia hydrogenotrophica* *Clostridium* spp. *Streptococcus* spp.	[136]
Propionate	GPR43 GPR41	Succinate pathway	*Bacteroides* spp. *Phascolarctobacterium succinatutens* *Dialister* spp. *Veillonella* spp.	[148]
		Acrylate pathway	*Megasphaera elsdenii* *Coprococcus catus*	[136]
		Propanediol pathway	*Salmonella* spp. *Roseburia inulinivorans* *Ruminococcus obeum*	[136]
Butyrate	GPR41 GPR109A	Butyrate kinase route	*Coprococcus comes* *Coprococcus eutactus*	[136]
		Bytyryl-CoA	*Coprococcus catus* *Eubacterium rectale* *Roseburia* spp. *Faecalibacterium prausnitzii*	[135,136]

#### 4.3.2. Bile Acids

BAs are steroid acids found predominantly in the bile of mammals and other vertebrates. When fat is present, the gut lumen secretes BAs. BAs come in two principal forms: cholic acid and chenodeoxycholic acid [149]. The gut microbiota transform primary BAs, which then interact with the Takeda-G-protein-receptor-5 (TGR-5) and the farnesoid X receptor (FXR) [150]. Through the FXR and TGR5, BAs exert impacts on metabolism [151]. According to Pedersen et al., the activation of the FXR and TGR5 enhances insulin sensitivity and glycogen production in the liver, raises pancreatic insulin output, and modulates brain satiety [150]. Taurocholic acid (TCA) is produced more often when animal fat is regularly consumed [151]. According to Agus et al., TCA prefers *Bilophila wadsworthia*, which is known to promote intestinal permeability and cause bacterial translocation. BA absorption may be hampered by this change in the microbiota. Consequently, there is a reduction in the expression of the FXR and FGF19, leading to an imbalance in BAs. Low-grade ongoing inflammation of the intestinal tract is related to this imbalance in BAs [151].

#### 4.3.3. Tryptophan

Tryptamine and indole derivatives are essential for maintaining the intestinal epithelium’s and immune cells’ homeostasis. According to Hendrikx et al., these substances are produced by the gut microbiome’s metabolism of tryptophan. These chemicals may encourage Th17 cells to reprogram into Treg cells, which would reduce inflammation. Nonetheless, a malfunction in the synthesis of the aryl hydrocarbon receptor ligand indole-3-propionic acid may result from a change in the host’s gut bacterial makeup, most likely brought on by food [152]. Due to this ligand’s malfunction, there is a reduction in GLP-1 and IL-22 release, breaking down intestinal permeability and causing LPS translocation and inflammation [151].

Microbial metabolites, such as SCFAs, secondary BAs, and tryptophan derivatives, play key roles in modulating immune responses. SCFAs, particularly butyrate, have been shown to enhance the production of Tregs and inhibit the activation of pro-inflammatory macrophages and dendritic cells [153]. Secondary BAs, produced by the microbial conversion of primary BA, can influence the differentiation and function of immune cells, further highlighting the intricate connection between microbial metabolism and immune regulation.

### 4.4. Dysbiosis and Inflammatory Conditions

Eubiosis, also known as “healthy microbiota”, is the state in which the microbiota in the intestines are in balance and has positive effects on the entire body. Stable functional cores of the microbiome, a high taxonomic diversity, and high microbial gene richness are characteristics of healthy gut microbial communities. Large changes in the proportions of the five main phyla of the gut microbiota bacteria or the emergence of new bacterial taxa define dysbiosis, an imbalance that promotes illness. The two main characteristics of dysbiosis are the outgrowth of Gram-negative lipopolysaccharide-producing bacteria and a decrease in microbial richness and diversity [116,154].

The causes of dysbiosis are multifactorial and can be influenced by genetic, environmental, dietary, and lifestyle factors [154]. An increased intestinal permeability is typically a sign of dysbiosis. Under healthy settings, Th1 and Th17 cells eliminate the translocation of a small number of bacterial products, such as the polysaccharides of *Bacteroides* spp. or mucosa-adherent segmented filamentous bacteria (SFB). Conversely, a high concentration of invasive bacteria causes the Toll-like receptors to become overactivated. This, in turn, causes an overexpression of inflammatory cytokines, which, in turn, causes epithelial damage and chronic inflammation [155]. Surprisingly, mice with a diabetic genotype were protected from developing the disease by higher SFB levels, as seen in MyD88-deficient mice models (an adaptor for various innate immune receptors which recognize microbial stimuli) [156]. This suggests that the microbiota can have both promotion and inhibition effects. Although a dysbiotic gut community may be the distinguishing feature of a number of inflammatory illnesses, dysbiosis may also act as a catalyst for the disruption of intestinal homeostasis and the emergence of inflammation [155].

An expanding range of ailments, including autoimmune diseases, neurological disorders, obesity, metabolic disease, and IBD, have been linked to dysbiosis in terms of both onset and severity. Additionally, it may act as the catalyst for both *Clostridium difficile*-associated diarrhea, which primarily affects elderly people, and necrotizing enterocolitis, which is seen in infants [116].

### 4.5. Association between Gut Microbiota and Inflammatory Diseases

According to Feng et al., the gut microbiota and their metabolites may control the inflammatory conditions of the host. Numerous studies have connected inflammatory illnesses to the gut microbiome [21]. According to Forbes et al., the gut microbiota are altered by immune-mediated inflammatory illnesses such as CD, UC, multiple sclerosis, and RA [157]. Furthermore, an abundance of studies have demonstrated the pathophysiology of the gut microbiota in inflammatory illnesses, including obesity, T1DM and T2DM, and asthma [158].

#### 4.5.1. Inflammatory Bowel Disease

IBD, encompassing CD and UC, is strongly associated with dysbiosis. Over 5 million people globally suffer from UC, the most prevalent kind of IBD [159,160]. Because of its mucous-layer-specific inflammation, the walls of the colon and rectum only sustain superficial damage [161]. Any portion of the gastrointestinal tract can be affected by CD, which is typified by erratic transmural inflammation that penetrates the intestinal wall into the serous layer and mostly affects the terminal ileum [124,161]. It has been shown in earlier research that abnormal intestinal permeability can predict the onset of IBD and is present in asymptomatic individuals years before the illness manifests [162]. Patients with IBD exhibit a reduced microbial diversity and altered composition of the gut microbiota, characterized by a decrease in beneficial bacteria such as *Faecalibacterium prausnitzii* and an increase in pathogenic bacteria like *Escherichia coli* [163].

These microbial shifts contribute to chronic intestinal inflammation through several mechanisms. Dysbiosis compromises the integrity of the gut barrier, leading to increased intestinal permeability, or “leaky gut”. This allows bacteria and their metabolites to translocate into the bloodstream, triggering immune responses and inflammation [164]. An altered gut microbiota composition can lead to inappropriate activation of the mucosal immune system. For instance, dysbiosis in IBD is associated with increased levels of pro-inflammatory cytokines, such as TNF-α and IL-6, which exacerbate inflammation [162]. Dysbiosis affects the production of microbial metabolites like SCFAs, which have anti-inflammatory properties. Reduced levels of SCFAs can contribute to inflammation and disease progression [5].

#### 4.5.2. Obesity and Metabolic Syndrome

Dysbiosis of the gut microbiota is also implicated in obesity and metabolic syndrome, conditions characterized by chronic low-grade inflammation. Obese individuals often have a less diverse gut microbiota and an altered ratio of F/B phyla, with a higher prevalence of Firmicutes [165]. This imbalance promotes metabolic inflammation through several pathways: dysbiosis can increase intestinal permeability, allowing LPS from Gram-negative bacteria to enter the circulation. This condition, known as metabolic endotoxemia, induces systemic inflammation and insulin resistance [166]. According to Ni et al., dysbiosis influences the inflammatory state of the adipose tissue. Certain gut bacteria produce metabolites that can modulate inflammation in the adipose tissue, contributing to insulin resistance and metabolic syndrome [163]. According to a study, obesity was linked to precancerous alterations in the transcriptome, while an elevated body mass index was linked to an increase in two proinflammatory colonic cytokines, TNF- α and IL6 [167].

#### 4.5.3. Rheumatoid Arthritis

RA is an autoimmune disease characterized by chronic inflammation and the destruction of cartilage and bones. It has been documented that the gut microbiota play a role in the development and progression of RA. Patients with RA often show an altered gut microbiota composition, with an increase in pro-inflammatory bacteria such as *Prevotella copri* [168]. The altered gut microbiota in RA can affect systemic immune responses, promoting the differentiation of pro-inflammatory T cells and the production of autoantibodies that target joint tissues. The concept of the gut–joint axis suggests that microbial antigens and metabolites can influence joint inflammation. Dysbiosis may lead to the production of inflammatory mediators that travel from the gut to the joints, exacerbating RA symptoms [168].

According to a Chinese study, *Lactobacillus salivarius* was more prevalent in the saliva, teeth, and stomach of RA patients. On the other hand, *Haemophilus* species declined in these patients across all investigated areas [155]. A reduced intestinal microbial diversity was also seen in RA patients in a different study, and this finding was connected with both antibody production and the length of the disease. According to Maeda and Takeda, RA patients had higher relative abundances of *Collinsella aerofaciens* and *Eggerthella lenta* and a lower relative abundance of *Faecalibacterium* [169]. The genus *Collinsella* has been shown in vitro to increase intestinal permeability and induce the expression of IL-17A. This suggests that the growth of these microorganisms in the human gut can lead to an increase in proinflammatory conditions and make them a potential cause of arthritis [170].

#### 4.5.4. Cardiovascular Diseases

CVDs, including atherosclerosis, have been associated with dysbiosis. Atherosclerosis is brought on by many risk factors, including metabolic syndrome, diabetes mellitus, tobacco use, and high blood pressure. Alongside these established risk factors for atherosclerosis, there is growing evidence to support the idea of a gut–systemic circulation axis in the atherosclerotic process. This axis is defined by the bloodstream passage of bacterially produced products such as LPS and TMAO [171]. The gut microbiota of patients with CVDs often show a reduced diversity and an increase in pro-inflammatory bacteria [172].

Certain gut bacteria metabolize dietary choline and carnitine into trimethylamine (TMA), which is further converted into TMAO in the liver. Elevated TMAO levels are associated with an increased risk of atherosclerosis and cardiovascular events. Dysbiosis can promote systemic inflammation and the activation of immune cells, contributing to the development and progression of atherosclerosis [172]. Numerous prospective studies have assessed the impact of low-grade endotoxemia on the risk of atherosclerosis, with the results showing that patients with high quantities of LPS are at a much higher risk [173]. It has also been noted that LPS contributes to the susceptibility of atherosclerotic plaques. Mice exposed to LPS displayed thrombus development and hemorrhaging. This result most likely stemmed from the creation of Leukotriene B4, a potent activator of leucocyte activation, and the activation of the arachidonic acid pathways, both of which are decreased in mice models of arterial inflammation [174].

The TMAO concentration has been suggested as a predictive and prognostic marker for CVDs due to the recent discovery of a positive correlation between it and acute coronary syndrome. Moreover, it has been documented that TMAO levels are correlated with the dimension of atherosclerotic plaques and the risk of cardiovascular events, including myocardial infarction, stroke, and mortality over three years. Through both inflammatory and metabolic processes, their levels have also been linked to unstable features of plaques, such as micro-vessels and a thinner fibrous cap [175].

#### 4.5.5. Type 2 Diabetes Mellitus

The acquired condition known as T2DM is characterized by an increase in cardiovascular risk and mortality, as well as systemic inflammation. In obesity, several gene variants and environmental variables are mutually associated with the etiology of T2DM. The reported elevated risk of T2DM in individuals undergoing complete colectomy provides indirect evidence of the microbiota’s role in T2DM development. As a result, resistance to diet-induced obesity has been observed in studies using germ-free mice; on the other hand, these mice showed an altered glucosidic tolerance and weight gain when exposed to bacteria specific to obesity, such as *Enterobacter cloacae*, or bacteria derived from obese donors [176].

AN increased intestinal permeability has been linked to T2DM, which may allow bacteria to pass through the gut barrier and cause low-grade systemic inflammation [177]. Remarkably, a long-term follow-up investigation revealed the prognostic significance of four bacterial species (*Clostridium citroniae*, *C. bolteae*, *Tyzzerella nexilis*, and *Ruminococcus gnavus*) in the development of T2DM. Numerous studies have shown altered gut microbiota in T2DM in the past, identifying a microbial signature for the illness and a connection between the gut microbiota and particular T2DM characteristics like insulin resistance [178].

A positive correlation has been observed in recent studies on diabetic patients between a few bacterial species and markers of systemic inflammation; specifically, the relative abundances of *Bifidobacterium adolescentis*, *Alistipes onderdonkii*, and *Eubacterium rectale* were positively correlated with IL-6, high-sensitivity C-reactive protein (CRP), and LPS-lipopolysaccharide binding protein; *Bacteroides thetaiotaomicron* was found to be positively correlated with the lipopolysaccharide-binding protein levels [179]. *Prevotella copri* and *Bacteroides vulgatus*, two bacteria that have been identified as being more prevalent in T2DM patients, have the ability to cause insulin resistance and enhance the availability of branched-chain amino acids in mice. *Ralstonia pickettii*-treated obese mice also demonstrated increased insulin resistance, pointing to a possible involvement of this bacterium in the development of T2DM [180].

#### 4.5.6. Allergic Diseases

Allergic diseases, such as asthma and atopic dermatitis, are linked to dysbiosis, particularly during early life. A reduced microbial diversity and an altered gut microbiota composition in infancy are associated with an increased risk of developing allergic diseases [150]. The gut microbiota play a crucial role in the development of the immune system. Dysbiosis can impair the maturation of regulatory T cells (Tregs) and skew immune responses towards a pro-allergic phenotype. Dysbiosis can compromise the integrity of the gut barrier, allowing allergens to penetrate and trigger immune responses that lead to allergic inflammation [181].

## 5. Diet and Inflammation

Diet significantly influences the levels of biomarkers of inflammation in the body, which can serve as indicators of the overall inflammatory state and risk for various chronic diseases. Different dietary patterns impact key inflammatory biomarkers such as CRP, IL-6, TNF-α, and others [182].

CRP is produced by the liver in response to inflammation. Elevated CRP levels are associated with an increased risk of CVD, diabetes, and other inflammatory conditions [183]. IL-6 is a cytokine involved in inflammation and infection responses. High levels of IL-6 are linked to chronic inflammatory diseases and conditions like obesity [184] and metabolic syndrome [185]. TNF-α is a pro-inflammatory cytokine that plays a key role in systemic inflammation. Elevated levels are associated with autoimmune diseases, insulin resistance [184], and CVD [186].

Adherence to the MD has been consistently associated with lower levels of CRP, IL-6, and TNF-α. This diet’s anti-inflammatory effects can be attributed to its high content of antioxidants, fiber, and healthy fats, particularly omega-3 fatty acids [42]. Rich in omega-3 polyunsaturated fats, the MD has anti-inflammatory and immunomodulatory effects by affecting the immune system function. These compounds decrease the production of pro-inflammatory factors like IL-1β, IL-6, TNF-α, VCAM-1, and MCP-1. They also lower ROS and nitrogen species levels while simultaneously increasing anti-inflammatory cytokines like IL-10 [187]. The MD pattern’s high fiber intake is another characteristic. It also benefits the intestinal microbiota by modifying their composition and encouraging the release of metabolites like SCFAs, which control immune functions (e.g., acetate, propionate, and butyrate) [188]. For instance, butyrate reduces the synthesis of IL-1β, TNF-α, NF-kB, and IL-12 to have anti-inflammatory effects. By encouraging the colonization of Bacteroidetes and specific advantageous *Clostridium* groups and reducing Proteobacteria and Bacillaceae phyla, following the MD also helps to restore microbiota eubiosis [2]. The effects of the MD, rich in extra virgin olive oil (HQ-EVOO), on overweight/obese participants and normal-weight controls were investigated by Luisi et al. The authors investigated the potential effects of a three-month HQ-EVOO-enriched MD on the gut microbiota composition, oxidative stress metrics, and inflammation. It is interesting to note that this dietary approach reduced IL-6, TNF-α, and myeloperoxidase (a marker of inflammation and endothelial dysfunction) in both research groups [189].

Vegetarian and vegan diets are associated with lower levels of inflammatory biomarkers, including CRP and IL-6. Studies have demonstrated that plant-based diets can reduce CRP levels, potentially due to their high fiber content, low saturated fat, and abundance of anti-inflammatory phytochemicals [190]. Considering that CRP is a well-established biomarker of systemic low-grade inflammation connected to a number of disorders, Menzel et al. suggested that adopting a vegetarian or vegan diet may reduce the levels of circulating inflammatory biomarkers and improve inflammatory processes. For vegan or vegetarian populations, these anti-inflammatory qualities may lower the risk of chronic inflammatory disorders [191]. Remarkably, recent research has demonstrated the function of inflammasomes in controlling the gut microbiota and gut homeostasis [192]. These are a collection of protein complexes that stimulate the release of pro-inflammatory cytokines and are capable of identifying a wide range of stimuli that cause inflammation. These processes could impact immunological homeostasis associated with a corresponding decrease in the likelihood of developing metabolic disorders, such as atherosclerosis or metabolic syndrome. The role of inflammasomes in regulating the gut flora offers a novel and promising research topic that may help us to understand the mechanisms by which the diet affects the gut microbiota, inflammation, and health, even though more research is obviously needed [191].

The WD, characterized by a high intake of processed foods, red meats, and refined sugars, is linked to elevated levels of CRP, IL-6, and TNF-α. Research shows that individuals consuming a WD have higher levels of systemic inflammation markers, contributing to an increased risk of chronic diseases [71]. The main alteration in the microbiota composition caused by a HFD in both animal and human models is a rise in the F/B ratio. According to Velazquez et al., the most prevalent phyla in the gut microbiota of mice fed with an LFD and animals fed with an HFD were Bacteroidetes and Firmicutes, which accounted for 61% and 32% of the gut microbiota in LFD mice and 73% and 21% in old-HFD mice, respectively. However, compared to the LFD animals, the F/B ratio was greater in the HFD mice [193]. Increases in the abundances of Bacilli, Clostridiales, and Erysipelotrichales—all members of the Firmicutes phylum—are said to be the primary cause of the observed alterations in the F/B ratio [194]. Moreover, in a recent study, Yang et al. found that a HFD drives colorectal tumorigenesis by inducing gut microbial dysbiosis, metabolomic dysregulation with elevated lysophosphatidic acid, and gut barrier dysfunction in mice [195]. On the other hand, *A. muciniphila* lowers the pathogen load in diets heavy in fiber, demonstrating the context-dependent advantages of this mucin specialist. Increased mucus penetrability and altered behaviors of *A. muciniphila* and other community members are the causes of the increased vulnerability to pathogens, not changes in host immune systems or pathogen responses [196].

Apart from a HFD, diets high in added sugars are associated with an increased production of pro-inflammatory biomarkers like CRP and IL-6. A clinical study by Kim et al. indicated that a high sugar intake elevates CRP levels and other markers of inflammation, highlighting the inflammatory potential of excessive sugar consumption [197]. In a mouse model, high-sugar diet consumption significantly increased the abundance of *Escherichia coli* in fecal samples and promoted gut inflammation and a systemic immune response [198]. Moreover, in other mice studies, high-sugar diets induced colon inflammation compared with a standard diet by altering the composition of the gut microbiota, increasing the levels of *Akkermansia muciniphila*, known to produce enzymes degrading the mucus layer [199]. Short-term exposure to a high-sugar diet in mice increases their susceptibility to colitis by reducing the production of SCFAs and increasing the gut permeability [200].

## 6. The Interconnected Triangle: Diet, Gut Microbiota, and Inflammation

### 6.1. Interconnection of Diet and Gut Microbiota

**Figure 3:**
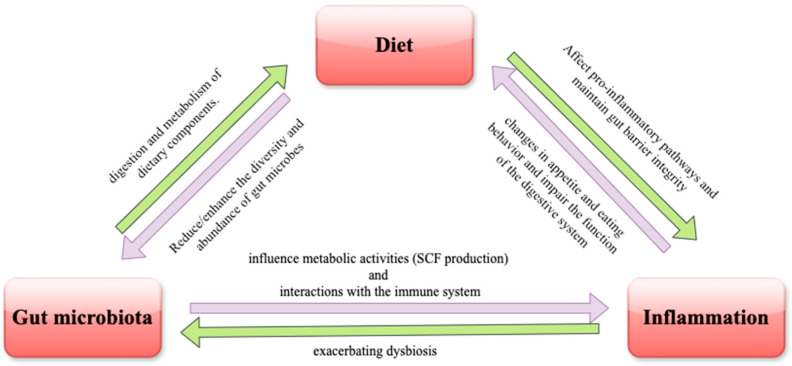
The interconnected triangle: diet, gut microbiota, and inflammation.

Diet is one of the most significant factors shaping the composition and function of the gut microbiota. Different dietary patterns, such as the WD, MD, and plant-based diets, have distinct effects on gut microbial communities. For instance, a WD high in fats and sugars promotes an imbalance known as dysbiosis, characterized by a reduction in beneficial bacteria and an increase in pathogenic bacteria. In contrast, diets rich in fiber and polyphenols, like the MD, enhance the diversity and abundance of beneficial microbes such as bifidobacteria and lactobacilli (Figure 3). These beneficial bacteria produce SCFAs and other metabolites that support gut health and reduce inflammation [2,60].

On the other hand, the gut microbiota play a crucial role in the digestion and metabolism of dietary components (Figure 3). Microbial enzymes break down complex carbohydrates, proteins, and fats that are indigestible by human enzymes alone. This breakdown results in the production of various metabolites, including SCFAs, which provide an energy source for colonocytes and influence the host metabolism [135]. Moreover, the gut bacteria synthesize essential vitamins such as vitamin K and B vitamins. The gut microbiota can also modulate appetite and eating behavior through the production of neurotransmitters and hormones, impacting dietary choices and nutrient intake [201].

### 6.2. Interconnection of Diet and Inflammation

Diet directly influences systemic inflammation through its impact on the gut microbiota and the immune system. Diets high in saturated fats and refined sugars promote pro-inflammatory pathways by increasing the production of endotoxins such as LPS [171] (Figure 3). Conversely, anti-inflammatory diets rich in omega-3 fatty acids, fiber, and polyphenols enhance the production of SCFAs, which have anti-inflammatory properties. These diets help to reduce the levels of pro-inflammatory cytokines and support the growth of beneficial bacteria that maintain the gut barrier integrity [5].

Chronic inflammation can alter dietary preferences and nutrient absorption. Inflammatory cytokines can affect the central nervous system, leading to changes in appetite and eating behavior, often resulting in a reduced food intake and altered taste preferences [77]. Inflammation can also impair the function of the digestive system, reducing the absorption of essential nutrients and leading to deficiencies that exacerbate inflammatory conditions (Figure 3). This cycle of inflammation and nutrient malabsorption can contribute to the progression of chronic diseases [164].

### 6.3. Interconnection between Gut Microbiota and Inflammation

Gut microbes significantly influence inflammatory processes through their metabolic activities and interactions with the immune system. Beneficial bacteria produce SCFAs, which enhance the gut barrier function, modulate immune responses, and inhibit the production of pro-inflammatory cytokines [144] (Figure 3). Dysbiosis, on the other hand, disrupts these processes, leading to an increased gut permeability and the translocation of bacterial endotoxins into the bloodstream. This endotoxemia triggers systemic inflammation and is associated with various inflammatory diseases such as IBD and RA [144,162].

On the other hand, chronic inflammation can negatively impact the gut microbiota, further exacerbating dysbiosis (Figure 3). Inflammatory conditions alter the gut environment, making it less hospitable for beneficial bacteria and promoting the growth of pathogenic microbes. This imbalance leads to a vicious cycle where inflammation begets dysbiosis and dysbiosis fuels further inflammation. Inflammatory diseases often show a reduced diversity of the gut microbiota, which is linked to worse clinical outcomes [157].

### 6.4. Role of the Host in the Triangular Relationship

In the triangular relationship between diet, the gut microbiome, and depression, the host plays a central role, with their genetic background, lifestyle factors, and environmental exposures significantly influencing this interaction. Variations in the abundance and composition of specific bacterial communities have been associated with specific genotypes of the host. Since genes affect the immune system function and prevalence of diseases, then certain alleles are associated with certain compositions. For instance, people with the rs651821 variant of the APOA5 gene tend to have more *Lactobacillus*, *Sutterella*, and *Methanobrevibacter*, which are associated with the risk of developing metabolic diseases [202]. According to the literature, specific variants of microbiome-associated genes are causally related to diseases including obesity, schizophrenia, Type 2 diabetes, amyotrophic lateral sclerosis, and inflammatory bowel disease [203]. Lifestyle factors, such as physical activity, stress levels, sleep patterns, and medication use (e.g., antibiotics and proton pump inhibitors), also impact the gut microbiome and its interaction with diet. Physical activity has been shown to promote gut microbiota diversity and SCFA production, while chronic stress can lead to dysbiosis by increasing the gut permeability and altering the microbial balance. Similarly, inadequate sleep and the use of medications can disrupt gut microbiota homeostasis, further complicating the relationship between diet and the microbiome [204]. Environmental exposures, including pollutants, toxins, and geographical factors, can modulate the gut microbiome and interact with both genetic predispositions and dietary factors. For instance, exposure to pollutants may exacerbate gut inflammation and disrupt microbial communities, which could amplify the effects of an unhealthy diet. Furthermore, factors such as urbanization, antibiotic use in food production, and water quality can have widespread effects on the microbiome compositions across populations, leading to variations in how individuals respond to dietary interventions [205]. Therefore, the host’s genetic background, lifestyle choices, and environmental context collectively influence the diet–microbiome–depression link, making it a complex and highly individualized relationship.

### 6.5. Impact of Diet, Gut Microbiota, and Inflammation on the Brain and Behavior

The gut–brain axis is a complex and bidirectional communication network that links the gastrointestinal system with the central nervous system, playing a crucial role in maintaining homeostasis and influencing behavior and mental health. This axis involves multiple pathways, including neural, hormonal, immune, and metabolic signaling, through which the gut microbiota can impact brain function and mood [206]. Furthermore, the host’s behavior is influenced by the relationship between the central nervous system and the enteric nervous system in the gut–brain axis. The enteric nervous system, sometimes called the “second brain”, is made up of two nerve plexuses, the myenteric and submucosal plexuses, glial cells, and motor and sensory neurons [207]. Therefore, mediators of microbiota–gut–brain communication regulated by bacterial metabolism include short-chain fatty acids (SCFAs), such as butyrate, neurotransmitters, such as serotonin and gamma-aminobutyric acid, hormones, such as cortisol, and immunomodulators. These pathways may be modified by changes in the gut microbiota, which may lead to the onset or exacerbation of neuropsychiatric diseases and accompanying symptoms [208].

A number of pathophysiological changes, including an altered mood and impaired stress responses, are caused by functional abnormalities in the gut–brain axis [209]. The vagus nerve modulates the relationship between psychobiotics and their associated psychophysiological effects, based on the results of multiple animal investigations. This is mostly because psychobiotics, whether given after vagotomy or after the vagus nerve has been severed, do not cause a physiological reaction [210].

Diet plays a pivotal role in shaping the composition and functionality of the gut microbiota, which, in turn, can influence the gut–brain axis. For example, diets rich in fiber promote the growth of beneficial bacteria that produce short-chain fatty acids (SCFAs). These SCFAs can cross the blood–brain barrier and modulate brain activity, contributing to anti-inflammatory effects and the production of neurotransmitters like serotonin, which are vital for mood regulation [Bai]. Conversely, diets high in fat and sugar can lead to gut dysbiosis, a state of microbial imbalance, which is associated with an increased gut permeability (“leaky gut”), systemic inflammation, and altered brain function. Such changes can contribute to the development of mental health disorders, including depression and anxiety [211].

Behavioral and psychological factors also play a significant role in this relationship. Stress, for example, can disrupt the gut microbiota, leading to changes in gut barrier function and promoting inflammation, which can further influence mood and cognitive function. Chronic stress is known to alter the gut microbiota composition, reducing the abundance of beneficial bacteria and increasing the presence of harmful bacteria, thereby exacerbating the gut–brain axis dysfunction [212].

Additionally, psychological factors like anxiety and depression can alter eating behaviors, which may further impact gut health. Emotional eating or a preference for unhealthy, high-fat, and high-sugar foods can perpetuate a vicious cycle, where a poor diet exacerbates gut dysbiosis, leading to further mental health decline. Understanding the intricate interplay between diet, the gut microbiota, and the gut–brain axis highlights the importance of a balanced diet and stress management in promoting both gut health and mental well-being [212].

## 7. Therapeutic Implications

The growing understanding of the interconnected relationship between diet, gut microbiota, and inflammation opens up promising avenues for therapeutic interventions aimed at managing various health conditions. These therapeutic implications can be harnessed to prevent and treat chronic inflammatory diseases, optimize gut health, and enhance overall well-being.

Fecal microbiota transplantation (FMT) has the potential to restore the complete microbiota ecosystem. To replace lost activities in the gut microbiota, single helpful strains or groups of them (probiotics) might be added; in the meantime, undesirable or hazardous strains could be eliminated with the use of bacteriophages, antibiotics, or antifungals. Lastly, it may be possible to inhibit or stop the synthesis of toxic metabolites or increase the synthesis of advantageous metabolites by targeting microbial metabolic pathways. FMT, which involves the transfer of stool from a healthy donor to a recipient with dysbiosis, has shown promise in treating recurrent *Clostridioides difficile* infection and has cure rates exceeding 90% [213]. FMT is being explored for other conditions like IBD and metabolic syndrome [214]. FMT has also been used experimentally to treat other gastrointestinal disorders, such as UC, constipation, IBS, liver diseases such as cirrhosis with encephalopathy and alcoholic hepatitis, and neurological diseases such as multiple sclerosis and Parkinson’s disease [201].

Probiotics are highly well-liked substances that affect the host health and gut microbiota. Probiotic microorganisms have a variety of actions that frequently cooperate. Their primary mechanisms include immune system modulation, enhanced intestinal barrier function, resistance to colonization, and metabolite synthesis that acts both locally (antimicrobials, enzymes, and organic acids) and remotely (hormones and neurochemicals). Strong evidence supports the safety and effectiveness of a number of probiotics, such as *Lactobacillus* species, *Bifidobacterium* species, and *Saccharomyces* species. Sanders et al. identified *Roseburia* spp. and *Faecalibacterium* spp. as further interesting options [215].

Promising therapeutic strategies also target the microbiome to either enhance the synthesis of protective metabolites or inhibit or reduce the production of harmful metabolites. One structural counterpart of choline that has been effectively used to prevent the microbial conversion of dietary choline to TMA is 3,3-dimethyl-1-butanol. Atherosclerosis and serious CVDs have been linked to TMAO, an oxidation product of TMA [201]. Remarkably, a new study indicates that, because of TMA’s nephrotoxicity and cardiotoxicity, it may be the primary offender rather than TMAO [216].

With the potential for personalized nutrition and tailoring dietary recommendations based on an individual’s unique gut microbiota composition, genetic profile, and specific health conditions, it is possible to achieve more effective and targeted outcomes. Personalized nutrition can help to manage conditions such as IBS, IBD, metabolic syndrome, and even mental health disorders like anxiety and depression. Advances in microbiome sequencing and bioinformatics tools facilitate the identification of specific microbial signatures associated with health and disease, allowing for the development of customized dietary interventions [217].

Probiotics can alleviate the symptoms of gastrointestinal disorders such as IBS and IBD, reducing bloating, abdominal pain, and diarrhea. Probiotics also play a role in enhancing immune responses by modulating the activity of immune cells and increasing the production of anti-inflammatory cytokines. Metabolic health can be improved through better glycemic control and lipid profiles, with certain probiotic strains aiding in weight management and reducing the risk of metabolic syndrome [8]. Moreover, emerging evidence suggests that probiotics can positively impact mental health by modulating the gut–brain axis, potentially alleviating symptoms of anxiety and depression [109]. Prebiotics support gut health by promoting beneficial bacterial growth, improving bowel regularity, and enhancing gut barrier function. They can reduce systemic inflammation and support immune homeostasis through the increased production of SCFAs [104].

However, the efficacy of probiotics and prebiotics is not without limitations. The benefits of probiotics are highly strain-specific, and not all strains provide the same health benefits, making it challenging to identify the most effective strains for specific conditions [218]. Additionally, probiotics must survive the harsh acidic environment of the stomach and bile salts to reach the intestines, where they must colonize effectively; otherwise, their benefits may be transient [219]. Individual variability, such as differences in existing gut microbiota, genetics, diet, and health status, also affects the efficacy of these treatments. Furthermore, the probiotic industry faces issues related to standardization, quality control, and regulatory approval, leading to variations in the potency, purity, and efficacy of probiotic products [219].

## 8. Research Gaps and Future Research Directions

Despite the growing body of evidence highlighting the importance of the diet–gut microbiota–inflammation axis, several research gaps remain. One significant gap is the lack of comprehensive, long-term studies that can conclusively establish causality rather than mere associations between diet, microbiota changes, and inflammation. Most existing studies are short-term and rely heavily on observational data, which can be influenced by numerous confounding factors. Furthermore, the individual variability in the gut microbiota composition and response to dietary interventions is not fully understood. This variability complicates the development of universal dietary guidelines and underscores the need for personalized nutrition strategies. Additionally, the mechanistic pathways through which specific dietary components influence microbial metabolites and subsequently modulate inflammatory responses are not completely elucidated. The interaction between different dietary components and their cumulative effects on the gut microbiota and inflammation also require further exploration.

To bridge these gaps, future research should focus on conducting long-term randomized controlled trials to establish causative relationships and understand the sustained impact of dietary interventions on the gut microbiota and inflammation. These studies should include diverse populations to account for genetic, environmental, and lifestyle factors that influence individual responses. Incorporating advanced technologies such as multi-omics (genomics, metabolomics, and proteomics) and machine learning can provide a more comprehensive understanding of the complex interactions within the diet–gut microbiota–inflammation axis. Detailed microbial and metabolic profiling before, during, and after dietary interventions can help to identify the key microbial players and metabolites involved in inflammatory processes.

Furthermore, studies should investigate the synergistic effects of combining various dietary components, such as fiber, polyphenols, and fermented foods, to determine the optimal dietary patterns for modulating the gut microbiota and reducing inflammation. Research into personalized nutrition should be intensified, utilizing individual microbiome data to tailor dietary recommendations and interventions. Exploring the role of lesser-studied bioactive compounds in foods, such as phytochemicals and micronutrients, and their specific effects on the gut microbiota and inflammation can also provide valuable insights.

Additionally, understanding the bidirectional relationship between the gut microbiota and diet, where the microbiota composition can influence dietary preferences and metabolic outcomes, is crucial. Investigating the impact of the gut microbiota on nutrient absorption and metabolism, and how this, in turn, affects inflammation, can provide a more holistic view of the triangular relationship. Finally, integrating behavioral and psychological aspects into research can help us to understand how diet and the microbiota influence mental health and vice versa, further elucidating the comprehensive impact of this interconnected triad on overall well-being.

As research advances in the fields of diet, the gut microbiome, and depression, there is a growing need for regulatory oversight to ensure the safety, efficacy, and ethical implementation of personalized interventions. Personalized nutrition and microbiome-targeted therapies hold great promise for improving mental health outcomes by tailoring dietary and therapeutic approaches to an individual’s unique microbiome composition and genetic background. However, without proper regulation, there is a risk of unsubstantiated claims, inadequate safety evaluations, and unequal access to these innovations.

Regulatory agencies must establish clear guidelines for developing, testing, and marketing microbiome-based therapies, including probiotics, prebiotics, and other dietary supplements. This includes enforcing rigorous clinical trials to demonstrate their effectiveness and monitoring long-term safety, particularly as interventions targeting the microbiome are still in their early stages. In parallel, ensuring that healthcare providers receive adequate training on the scientific basis and potential risks of these interventions is crucial to avoid the misuse of and overreliance on emerging but unproven therapies.

Equitable access to personalized interventions is another critical issue. Personalized nutrition and microbiome-targeted treatments often require advanced diagnostic tools, such as genetic sequencing and microbiome profiling, which can be costly and may not be readily available to all individuals, particularly those from low-income or underserved populations. This could exacerbate existing health disparities by making these promising treatments accessible only to those who can afford them. Policymakers must consider strategies to make these interventions more widely available, such as subsidizing costs, integrating them into public health programs, and supporting research that focuses on diverse populations. Therefore, a combination of robust regulatory oversight and proactive measures to promote equitable access is essential for realizing the full potential of personalized interventions in managing depression and other health conditions linked to the gut microbiome.

## 9. Conclusions

The intricate relationship among diet, the gut microbiota, and inflammation underscores the importance of dietary choices in maintaining health and preventing chronic diseases. Evidence suggests that dietary patterns significantly influence the composition and functionality of the gut microbiota, which, in turn, play a crucial role in modulating inflammatory processes. Beneficial dietary components, such as fiber, polyphenols, and healthy fats, support a diverse and balanced gut microbiota, promoting anti-inflammatory pathways and overall health. Conversely, diets high in saturated fats and refined sugars can lead to dysbiosis and increased inflammation, contributing to the development and progression of chronic diseases. Therapeutic strategies, including probiotics, prebiotics, personalized nutrition, and fecal microbiota transplantation, hold promise in modulating the gut microbiota to reduce inflammation and improve health outcomes. Future research should continue to explore the mechanistic pathways of this triad, aiming to develop targeted interventions that harness the power of the gut microbiota for disease prevention and management. Understanding and leveraging the interconnected triangle of diet, the gut microbiota, and inflammation could pave the way for innovative approaches to enhance human health and treat chronic inflammatory conditions.
